# Mechanistic Advances in the Therapeutic Application of Bixin for Lung Inflammation In Vitro and In Vivo

**DOI:** 10.3390/ph18040530

**Published:** 2025-04-05

**Authors:** Alexsandro Tavares Figueiredo-Junior, Bruno Clemente Brandão Marques, Douglas Galdino dos Santos, Wesley Leandro Gouveia, Raysa Magali Pillpe Meza, Luzineide Wanderley Tinoco, Lídia Moreira Lima, Samuel Santos Valenca, Manuella Lanzetti

**Affiliations:** 1 Programa de Pós-Graduação em Farmacologia e Química Medicinal, Instituto de Ciências Biomédicas, Centro de Ciências da Saúde, Universidade Federal do Rio de Janeiro, Cidade Universitária, Rio de Janeiro 21941-902, RJ, Brazil; figueiredojunior.at@gmail.com (A.T.F.-J.); bruno.cbmarques@gmail.com (B.C.B.M.); dgaldino@ufrj.br (D.G.d.S.); wleandrogouveia@gmail.com (W.L.G.); rpillpe@gmail.com (R.M.P.M.); lwtinoco@ippn.ufrj.br (L.W.T.); lidialima@icb.ufrj.br (L.M.L.); manuellalanzetti@icb.ufrj.br (M.L.); 2 Laboratório de Avaliação e Síntese de Substâncias Bioativas (LASSBio^®^), Instituto Nacional de Ciência e Tecnologia de Fármacos e Medicamentos (INCT-INOFAR), Centro de Ciências da Saúde, Universidade Federal do Rio de Janeiro, Cidade Universitária, Rio de Janeiro 21941-971, RJ, Brazil; 3 Instituto de Pesquisas de Produtos Naturais, Centro de Ciências da Saúde, Universidade Federal do Rio de Janeiro, Cidade Universitária, Rio de Janeiro 21941-902, RJ, Brazil

**Keywords:** Nrf2 pathway, oxidative stress, bixin, potassium bixinate, antioxidant, inflammation

## Abstract

**Background:** Nrf2 plays a key role in regulating the antioxidant response against oxidative stress. Therefore, it is imperative to examine the advantages of Nrf2 activation by new small molecules capable of inhibiting the Nrf2-Keap1 protein interaction that do not present electrophilic sites, since electrophilic compounds have intrinsic toxicity. The bixin pigment has been used as a form of treatment and prevention of several pathological conditions in animal models since it was described as an Nrf2 activator without electrophilic sites. This study aims to synthetize a soluble derivate KBx (potassium bixinate) and evaluate its ability to activate Nrf2/ARE in a model of exposure to cigarette smoke extract (CSE; in vitro) and intranasal LPS administration (in vivo). **Methods:** In the in vivo study, C57BL/6 mice were pretreated with 200 mg/kg of KBx (gavage) during 5 consecutive days and then challenged with 60 µg of LPS i.n. for 16 h. Bronchoalveolar lavage was collected to examine cytokines dosage. In the in vitro study, RAW 264.7 macrophages were exposed to CSE and post-treated with KBx to evaluate their ability to revert the redox imbalance caused by the stressor. **Results:** KBx was characterized using mass spectrometry (433.1778 *m*/*z*). KC levels were increased in the LPS group (*p* = 0.021), and KBx inhibited this (*p* = 0.001). IL-10 levels were decreased (*p* = 0.055) in the LPS group that was prevented when pretreated with KBx (*p* = 0.037). The in vitro study showed KBx to be a more potent derivate of bixin through its ability to intercept ROS formation with three-fold more potency, and it showed an anti-inflammatory propriety by reducing the nuclear translocation of p65 (*p* < 0.001). **Conclusions:** In conclusion, these data suggest that KBx was able to activate the Nrf2/ARE pathway and intercept ROS formation induced by CSE and LPS in both in vivo and in vitro studies.

## 1. Introduction

*Bixa orellana* L., commonly known as annatto, is a tropical plant renowned for its carotenoid-rich seeds, particularly bixin, which constitutes approximately 80% of its carotenoid content [1]. Bixin exhibits potent antioxidant properties, effectively neutralizing free radicals and oxidative agents. However, its hydrophobic nature presents significant challenges regarding solubility and bioavailability, limiting its therapeutic applications [2]. Annatto seed extracts have been investigated for their potential in treating and preventing various health conditions in animal models, including low-density lipoprotein (LDL) oxidation [3], hepatocarcinoma [4], diabetes [5], skin disorders [6], lung injury [7], metabolic syndrome [8], and cardiac injury [9]. These benefits are generally attributed to the extract’s ability to combat oxidative stress. Bixin is highly effective at neutralizing and deactivating free radicals and non-radical oxidants, exhibiting a significant scavenging effect [10]. However, bixin’s extensive carbon chain renders it highly hydrophobic, which limits its bioavailability and poses challenges for developing pharmaceutical formulations and in vivo applications [11].

In prior research, bixin was tested in the form of polymeric nanoparticles in an in vivo murine model of acute lung inflammation (ALI) induced by cigarette smoke (CS) exposure [2,7]. The results were promising and provided a foundation for further investigation into bixin using alternative approaches to improve its production and viability. To address these limitations, medicinal chemistry has explored various strategies, including the formation of ionic salts to enhance solubility. CS contains a variety of free radicals and oxidants, including alkyl, peroxyl, nitric oxide, and superoxide anion radicals, as well as semiquinone-derived compounds in its particulate matter [12]. Exposure to these substances can activate alveolar macrophages and recruit neutrophils, which then prompt both resident and infiltrated leukocytes to generate reactive oxygen species (ROS) and inflammatory mediators [13]. This process contributes to lung injury, partly due to the disruption of redox balance [14]. Oxidative stress leads to cellular damage, including tyrosine nitration of proteins [15], apoptosis through lipid peroxidation and caspase activation [16], and increased leukocyte recruitment to the lung parenchyma by enhancing the expression of cytokines such as tumor necrosis factor alpha (TNF-α) [17].

The formation of salts from lipophilic compounds involves the reaction between an ionizable site within an apolar molecule and an acid or base [18]. This process yields a salt, resulting in the formation of positively and negatively charged ions. The resulting salt improves solubility in polar solvents due to enhanced hydrogen bonding between the salt molecules and the solvent [19]. Beyond solubility enhancement, salt formation can modulate, indirectly, other critical pharmacokinetic and pharmacodynamic properties, including chemical stability and molecular target interactions [20]. In summary, this research aims to enhance the therapeutic potential of bixin through the development of potassium bixinate, addressing its solubility issues and evaluating its efficacy against oxidative and inflammatory stimuli. By combining chemical innovation with biological evaluation, this study seeks to provide new insights into the therapeutic applications of annatto-derived compounds.

## 2. Results

### 2.1. Characterization of Bixin Z-Diastereomer

**Figure 1:**
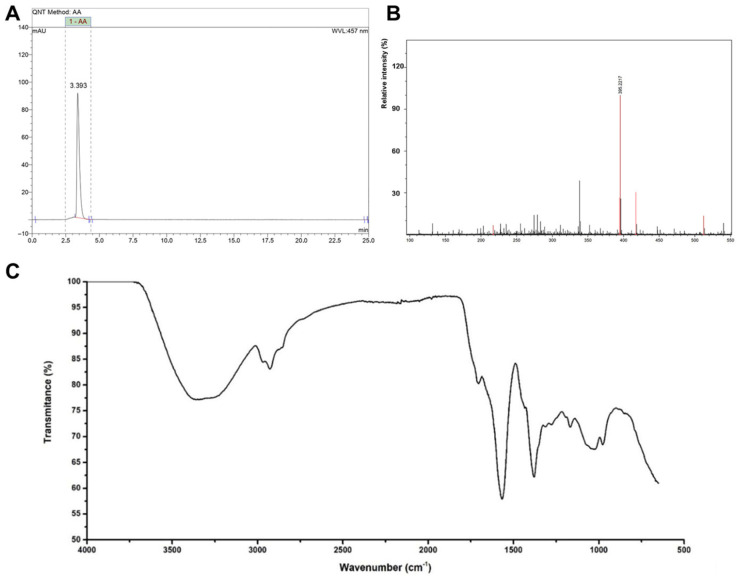
Bixin characterization. (**A**) Chromatogram of purified bixin acquired in a LC-20AD Shimadzu (Kyoto, Japan) equipped with a 100-5C18 Kromasil (Bohus, Sweden) analytical column (4.6 mm × 250 mm, 5 μm), detection performed with SPD-M20A Shimadzu (Kyoto, Japan) detector set at 457 nm wavelengths in an isocratic mobile phase of 85% acetonitrile and 15% methanol at 22 °C with a flow rate of 1 mL/min. (**B**) Mass spectrometer spectrum of bixin acquired in an electrospray ionization spectrometer, amaZon SL model Esquire 6000—ESI Ion Trap Msn System Bruker (Billerica, MA, USA). (**C**) FTIR spectrum of bixin acquired in a Nicolet iS10 FTIR Spectrometer (ThermoFisher Scientific, Waltham, MA, USA) in a range from 400 to 4000 cm^−1^.

The chromatogram shown in Figure 1A displays a single peak at 3.320 min at 457 nm, with a total run time of 25 min. The sample was subsequently analyzed using mass spectrometry with positive electrospray ionization modes, with the results presented in Figure 1B. The base peak corresponded to a structure with an *m*/*z* value of 395.22. The FTIR spectrum (Figure 1C) revealed key absorption peaks at 727 cm^−1^, 964 cm^−1^, 1159 cm^−1^, 1379 cm^−1^, 1431 cm^−1^, 1608 cm^−1^, 1717 cm^−1^, 2853 cm^−1^, and 2922 cm^−1^, along with a broad band between 3070 cm^−1^ and 3641 cm^−1^, with a minimum at 3426 cm^−1^. These data support the obtaining of purified HBx.

**Figure 2:**
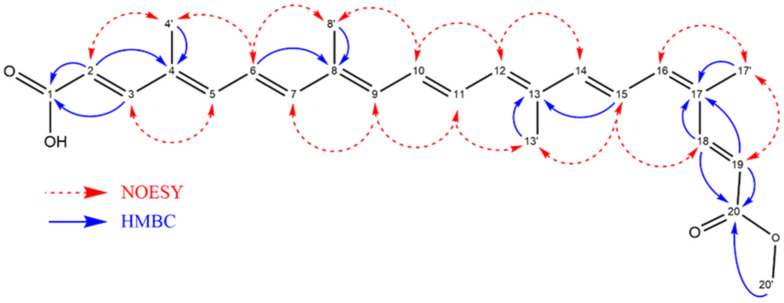
Key HMBC and NOESY correlations observed for the isolated bixin. The HMBC (blue arrows) indicate correlations between hydrogen and carbon atoms through two or three bonds. The NOESY (red dashed arrows) correlations show spatial proximities between hydrogens. These correlations contribute to the structural elucidation of (2E,4E,6E,8E,10E,12E,14E,16Z,18E)-20-methoxy-4,8,13,17-tetramethyl-20-oxoicosa-2,4,6,8,10,12,14,16,18-nonaenoic acid.

The NMR experiments were conducted to validate the proposed structure (Supplementary Figures S1–S5). In the polyene region, the ^1^H NMR spectra displayed signals at δH 7.89 (1H, d, *J* = 15.4 Hz, H19), δH 7.27 (1H, d, *J* = 15.5 Hz, H3), δH 5.95 (1H, d, *J* = 15.4 Hz, H19), and δH 5.83 (1H, d, *J* = 15.5 Hz, H2), along with overlapping resonances integrating for ten protons in the δH 6.41–6.92 range. A singlet at δH 3.70, integrating for three protons, was assigned to the methyl ester group. Additionally, a doublet of doublets in δH 1.96 were attributed to the protons of the methyl groups attached to the polyunsaturated chain. Compared to all-E-bixin or all-Z-bixin, the disruption of symmetry in the chemical shifts is attributed to the presence of a single Z double bond at C16–C17 and the substitution of a carboxylic acid group at position C1 with an ester in C20. The correlations observed in the HMBC and COSY spectrum unequivocally confirmed the assignments of H16 (δH 6.51), H17′ (δH 1.94), H4′ (δH 1.93), and H5 (δH 6.63) signals. The signal of H16 appeared slightly more shielded than H5, which is located near the acid function. Previous chemical studies have also identified the 16-Z-bixin isomer as the most abundant in annatto seeds [21], although, when bixin is isolated, the thermodynamically unstable (Z)-isomer undergoes isomerization, transforming the molecule into the all-E-bixin form (see ACS https://www.acs.org/molecule-of-the-week/archive/b/bixin.html, accessed on 13 March 2025). Meanwhile, NOESY correlations (Supplementary Figure S5) revealed spatial proximities between hydrogen atoms, which were instrumental in assigning the stereochemistry of the Z-bixin isomer. Notably, the interaction between δH 1.94 (H17′) and 6.51 (H16) supported a Z configuration at the C16–C17 double bond. Collectively, these NMR data conclusively established the compound as the Z-bixin isomer, with a (2E,4E,6E,8E,10E,12E,14E,16Z,18E) configuration. Detailed signal assignments and correlations are summarized in Supplementary Table S1, while key HMBC and NOESY interactions are highlighted in Figure 2.

**Figure 3:**
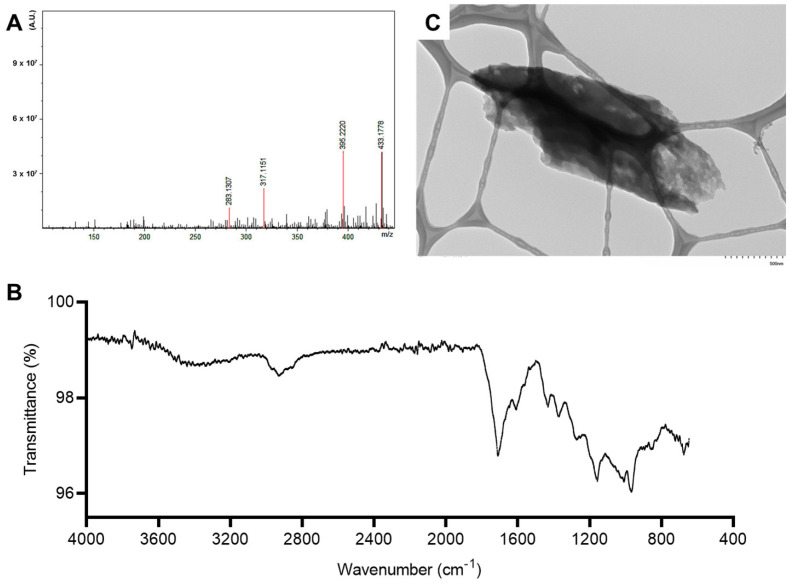
Potassium bixinate characterization. (**A**) Mass spectrometer spectrum of bixin acquired in an electrospray ionization Fourier transform ion cyclotron resonance mass spectrometry ESI Ion Trap Bruker (Billerica, MA, USA). (**B**) FTIR spectrum of bixin acquired in a Nicolet iS10 FTIR Spectrometer (ThermoFisher Scientific, Waltham, MA, USA) in a range from 400 to 4000 cm^−1^. (**C**) Transmission electron microscopy (TEM) image of KBx acquired using a HT7800 HITACHI (Tokyo, Japan) microscope at 100 kV in a magnification of 60,000×.

For KBx, samples were further analyzed using positive electrospray ionization mass spectrometry, FTIR, and TEM. The base peak corresponded to a structure with a *m*/*z* of 433.1778, although a peak with a very similar intensity at *m*/*z* 395.2220 was also detected (Figure 3A). The FTIR spectrum shown in Figure 3B reveals major peaks at wavenumbers 1172 cm^−1^, 1421 cm^−1^, 1557 cm^−1^, 1692 cm^−1^, and 2851 cm^−1^, and the absence of a broad band between 3000 and 3600 cm^−1^. The TEM images demonstrate that a well-defined crystal lattice did not form, likely due to the presence of irregular edges and a partially amorphous structure, indicative of a low degree of crystallinity (Figure 3C).

### 2.2. Bixin Has Low Solubility, Limited Intestinal Permeability, and High Stability

**Figure 4:**
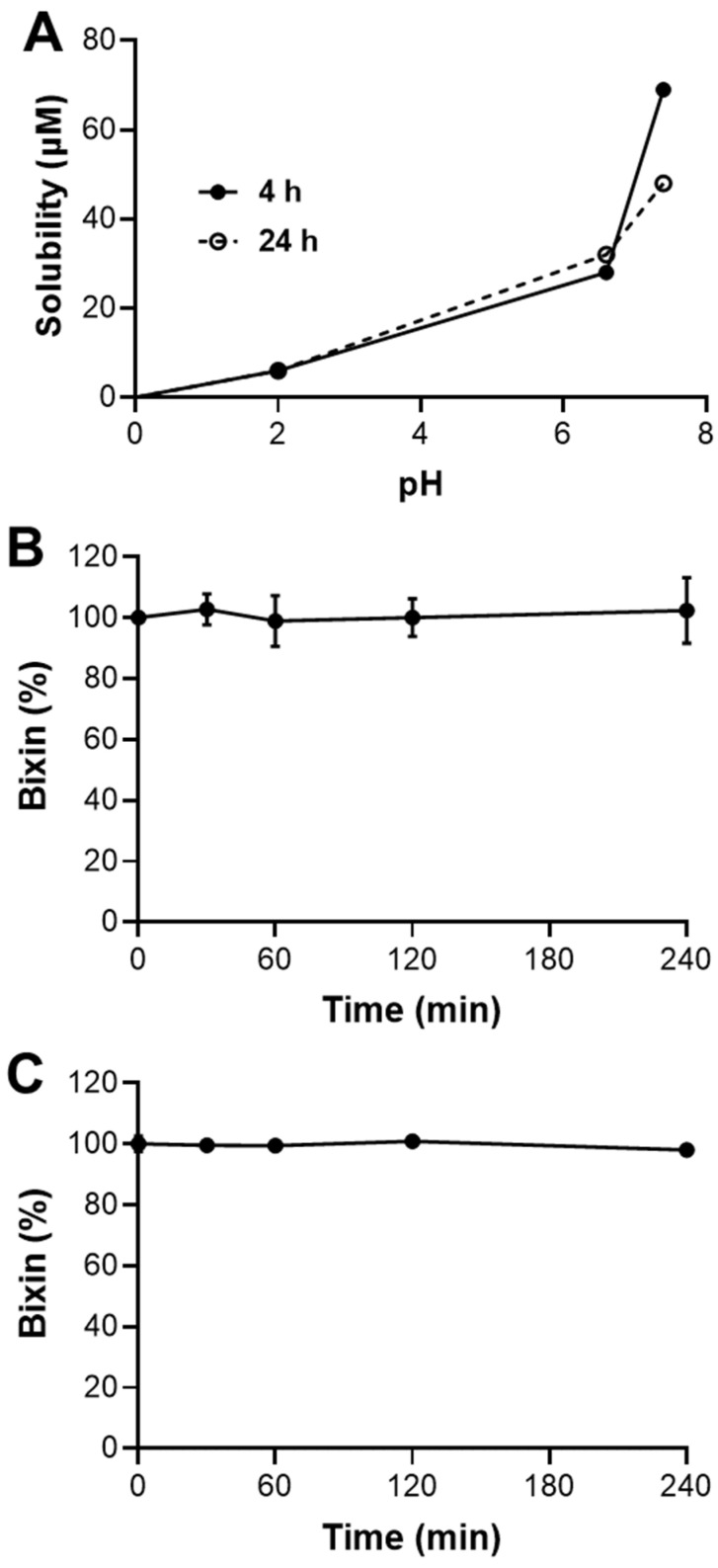
Drug-like profile of bixin. (**A**) In a solubility profile of bixin’s time- and pH-dependence, bixin was assessed in pH 2.0, 6.6, and 7.4 at 4 and 24 h. (**B**) Metabolic stability of bixin in rat plasma. The curve of sample concentration vs. incubation time shows the concentration of bixin; calculations were made from the natural logarithm of the sample concentration vs. incubation time, using, as the internal standard, biphenyl-4-carboxylate methyl. (**C**) Metabolic stability of bixin in rat liver microsomes in the absence cofactors (NADPH generating system). The curve of sample concentration vs. incubation time shows the concentration of bixin; calculations were made from the natural logarithm of the sample concentration vs. incubation time, using, as the internal standard, bi-phenyl-4-carboxylate methyl. All data are represented as the average of triplicate incubations. Results are expressed as the means of three independent experiments.

The solubility of bixin was evaluated at different pH levels (2.0, 6.6, and 7.4—Figure 4A) over time intervals of 4 h and 24 h. At pH 2.0, bixin’s solubility remained below 1 μM at both 4 and 24 h, indicating minimal solubility under acidic conditions. At pH 6.6, the solubility increased to 30 μM after 4 h, but slightly decreased to 11 μM after 24 h. The highest solubility was observed at pH 7.4, where bixin reached 64 μM after 4 h and decreased to 43 μM after 24 h. Bixin’s plasmatic stability was assessed over 4 h (Figure 4B). The compound demonstrated high stability in plasma, with no significant degradation observed during the experiment. Bixin retained nearly 100% of its initial concentration, indicating that it remains stable in plasma over time. Microsomal stability tests were conducted in the absence of cofactors to evaluate the metabolic hydrolysis stability of bixin (Figure 4C). Similarly to the plasmatic stability results, bixin exhibited high stability in microsomes, with negligible degradation over a period of 4 h. The compound maintained nearly 100% of its initial concentration, suggesting its resistance to enzymatic degradation in microsomes.

In the PAMPA-GIT assay (Table 1), HBx exhibited a permeability coefficient (Pe) of 0.36 × 10^−6^ cm/s and an absorbed fraction (Fa%) of 12.79%. These values classify HBx as having low intestinal permeability, suggesting that its absorption through the gastrointestinal tract is limited. The low Fa% indicates that HBx may face challenges in achieving effective absorption when administered orally.

### 2.3. Effects of Bixin and Potassium Bixinate on Cigarette Smoke Extract in RAW 264.7 Macrophages

**Figure 5:**
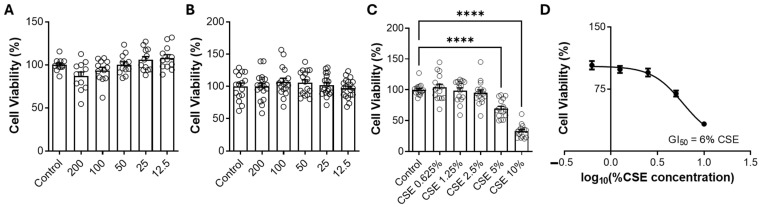
Evaluation of cell viability in RAW 264.7 cells treated with HBx, KBx, and CSE. (**A**) Effect of HBx (200–12.5 μM) on cell viability. (**B**) Effect of KBx (200–12.5 μM) on cell viability. (**C**) Effect of different concentrations of cigarette smoke extract (CSE, 0.625–10%) on cell viability. (**D**) Determination of CSE GI50 (6%) for cell viability using a dose–response analysis (log10[%CSE]). Results are presented as mean ± SEM of five different experiments. **** *p* < 0.0001.

An MTT assay was performed to evaluate the viability of RAW 264.7 macrophages stimulated with CSE and treated with HBx or KBx. The MTT salt is metabolized by mitochondria into an insoluble formazan-derived metabolite that precipitates at the bottom of the plate wells. This allows for extrapolation of the data to determine the density of viable cells in each well. Figure 5 presents the results of this assay, showing the effects of HBx, KBx, and CSE after 3 h of exposure. Figure 5A,B shows that treatment with HBx or KBx at concentrations ranging from 12.5 to 200 μM did not affect cell viability, indicating any cytotoxic effects under these conditions. Figure 5C indicates that CSE exhibits cytotoxic activity at concentrations of 5% and above, with a reduction of approximately 60% in cell viability at a concentration of 10%. By extrapolating these data to a concentration–response curve (Figure 5D), the GI50 (concentration causing a 50% reduction in cell viability) was calculated to be 6%.

### 2.4. Bixin and Potassium Bixinate Act as ROS Scavengers in RAW 264.7 Induced by LPS

**Figure 6:**
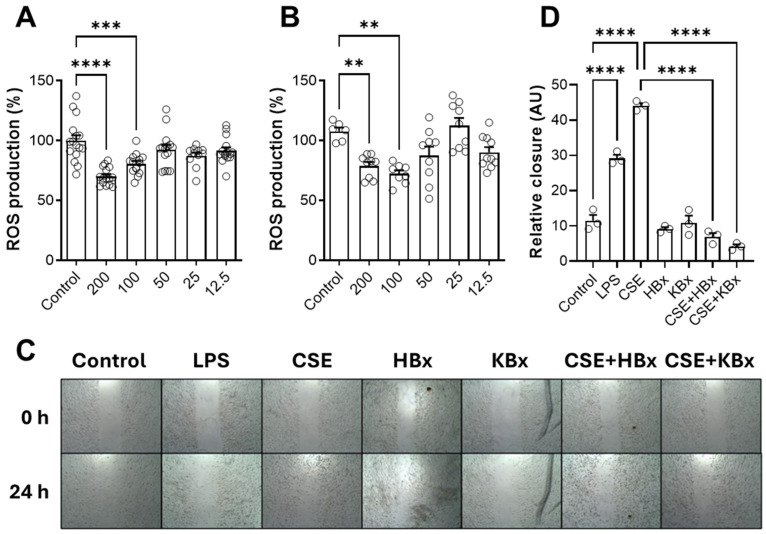
Effects of HBx, KBx, and CSE on ROS production and cell migration in RAW 264.7 cells. (**A**) Evaluation of ROS production induced by LPS and the scavenging activity of HBx at different concentrations (200–12.5 μM). (**B**) Evaluation of ROS production induced by LPS and the scavenging activity of KBx at different concentrations (200–12.5 μM). (**C**) Representative images of the wound healing assay at 0 and 24 h under different treatment conditions, magnification 10×. (**D**) Quantitative analysis of wound healing (wound closure percentage) after 24 h. Results are presented as mean ± SEM of three different experiments. ** *p* < 0.01, *** *p* < 0.001, and **** *p* < 0.0001.

RAW 264.7 cells were stimulated with LPS (10 μg/mL) and simultaneously treated with various concentrations of HBx or KBx to evaluate their activity in inhibiting ROS production over 3 h. Figure 6 shows the results for HBx (A) and KBx (B). Both compounds demonstrated a dose-dependent reduction in ROS production, starting from 100 µM concentration.

### 2.5. Bixin and Potassium Bixinate Inhibit RAW 264.7 Cell Migration Induced by Cigarette Smoke Extract

A wound healing assay was conducted to evaluate the effects of HBx and KBx under cell migration. RAW 264.7 cells were stimulated with CSE (5%), while LPS (10 μg/mL) was used as a positive control for migration. After 24 h, cell migration was quantified. Since the cells were pretreated with mitomycin C (an antiproliferative agent), it can be confirmed that the wound closure observed was due to actual migration and not cell proliferation. Figure 6C displays representative images captured at 0 and 24 h after the stimulus, while Figure 6D shows the corresponding quantification. The control group, treated only with mitomycin C without any stimulus, exhibited approximately 10% wound closure. In contrast, the positive control (LPS) and CSE groups demonstrated higher values, indicating that these stimuli promoted cell migration to close the wound, with the CSE group showing a migration rate nearly 50% higher than LPS. When cells were treated individually with 200 μM of HBx or KBx, no migration occurred, making them comparable to the negative control group. Additionally, when cells were exposed to CSE and treated with both HBx and KBx, the cell migration observed in the CSE group was completely inhibited, restoring the migration levels to those of the control group.

**Figure 7:**
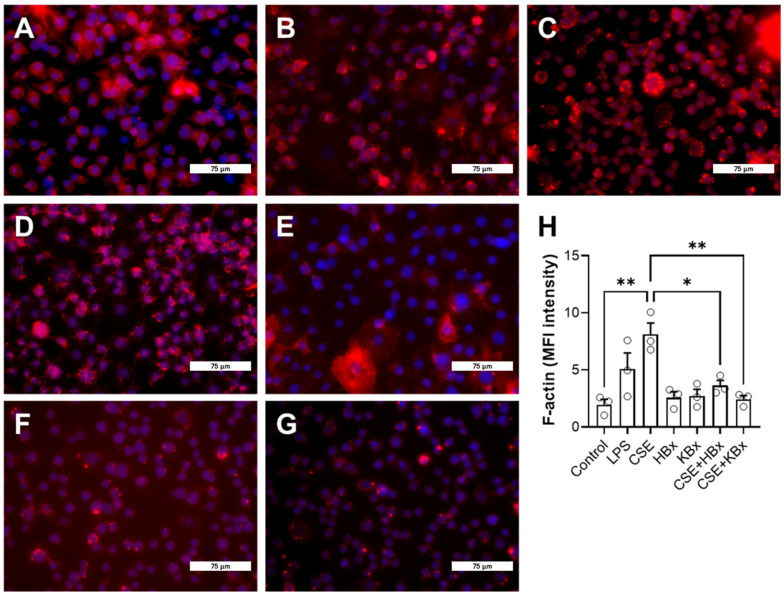
Bixin and potassium bixinate modulate the polymerization of actin filaments in RAW 264.7 cells in response to cigarette smoke extract. (**A**) control group, (**B**) LPS (10 μg/mL), (**C**) 5% CSE, (**D**) 200 μM HBx, (**E**) 200 μM KBx, (**F**) CSE + HBx, (**G**) CSE + KBx, and (**H**) quantification of mean fluorescence intensity (MFI). The figure represents a fluorescence microscopy for F-actin (red) and DAPI (blue) and all images are at 40× magnification, scale bar 75 μm. Results are presented as mean ± SEM of three different experiments. * *p* < 0.05 and ** *p* < 0.01.

Phalloidin and rhodamine staining were performed to visualize polarized actin filaments (F-actin, red) and cell nuclei (DAPI, blue) (Figure 7). The groups analyzed were as follows: (A) control, (B) LPS-exposed cells (10 μg/mL), (C) cells exposed to 5% CSE, (D) HBx-only, (E) KBx-only, (F) CSE + HBx, and (G) CSE + KBx. The quantification of mean fluorescence intensity (MFI) is shown in Figure 7H. Both LPS and CSE significantly increased F-actin labeling, while the HBx and KBx treatments maintained F-actin at control levels, even in the presence of CSE.

### 2.6. Bixin and Potassium Bixinate Prevent p-p65 NF-κB Nuclear Translocation in RAW 264.7 Cells Stimulated by Cigarette Smoke Extract

**Figure 8:**
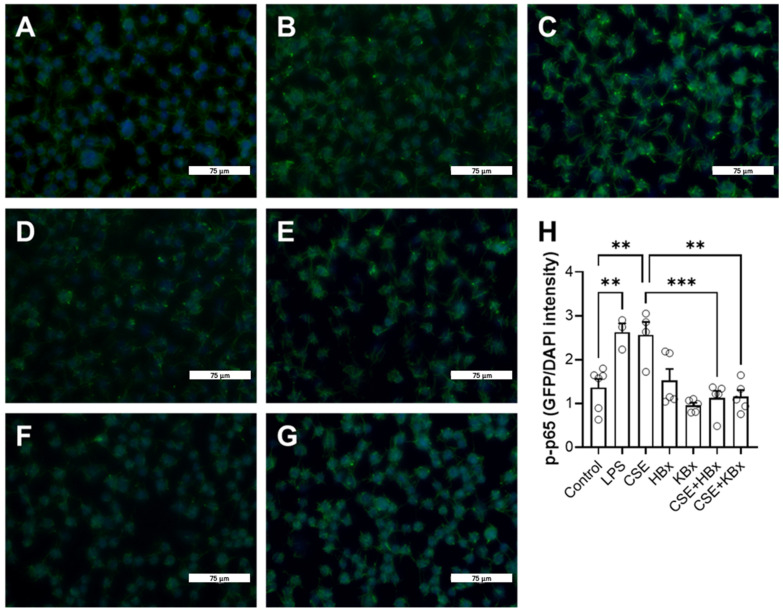
Bixin and potassium bixinate reduce the nuclear translocation of p-p65 in the face of cigarette smoke extract stimulus in RAW 264.7 cell. Immunocytochemistry for phosphorylated p65 subunit of NF-κB, green: p-p65, blue: DAPI. (**A**) control group, (**B**) LPS (10 μg/mL), (**C**) 5% CSE, (**D**) 200 μM HBx, (**E**) 200 μM KBx, (**F**) CSE + HBx, (**G**) CSE + KBx, and (**H**) quantification of MFI of GFP per DAPI. All images are at 40× magnification, scale bar 75 μm. Results are presented as mean ± SEM of three different experiments. ** *p* < 0.01 and *** *p* < 0.001.

Immunostaining for the p-p65 subunit of the transcription factor NF-κB was performed in RAW 264.7 cells. In Figure 8, the control group shows blue nuclear staining with DAPI (Figure 8A), while green p-p65 fluorescence is predominantly localized in the cytoplasm. Qualitatively, in the CSE (5%) and LPS (10 μg/mL) groups, a higher intensity of green fluorescence is observed throughout the cell, including the nucleus, where double labeling (green and blue) is evident (Figure 8B and 8C, respectively). In contrast, the groups exposed only to HBx or KBx (200 μM) do not display a significant increase in green fluorescence intensity colocalized with the blue DAPI staining (Figure 8D,E), similar to the control group. This pattern is also observed in the groups exposed to CSE and subsequently treated with HBx (Figure 8F) or KBx (Figure 8G), where green fluorescence colocalized with blue remains minimal. The graph in Figure 8H quantifies the average green fluorescence intensity relative to blue fluorescence, representing the degree of colocalization. This quantification confirms the visual observations from the images, indicating the presence of p-p65 in the nucleus and supporting the conclusion that HBx and KBx prevent p-p65 NF-κB nuclear translocation in cells stimulated with CSE.

### 2.7. Bixin and Potassium Bixinate Modulate Inflammatory Pathways by Regulating HDAC6, ICAM-1, and NOS2 Expression in RAW 264.7 Cells

**Figure 9:**
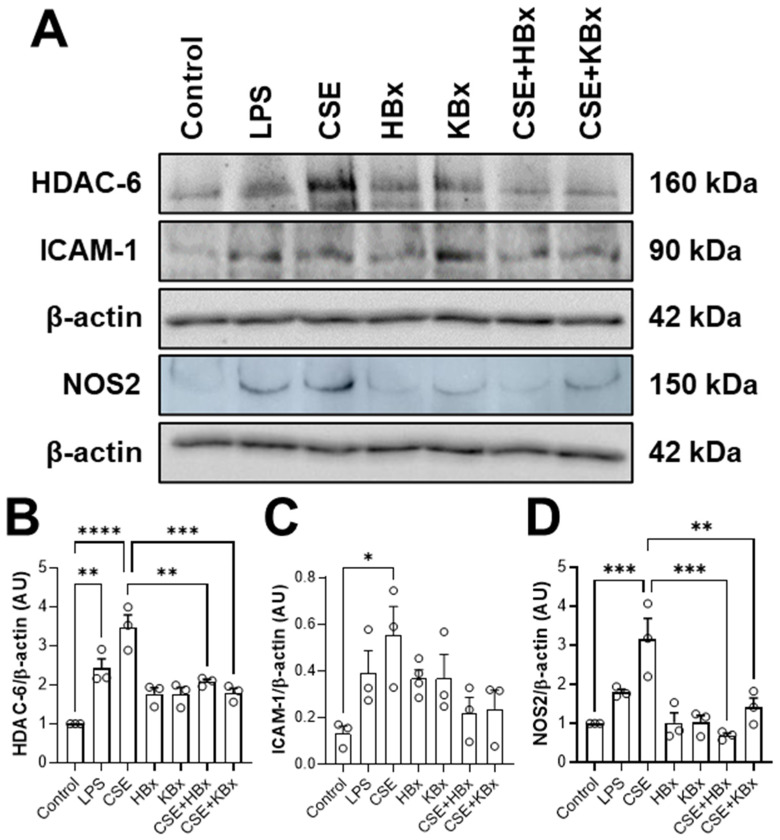
Effects of bixin and potassium bixinate in HDAC6, ICAM-1, and NOS2 protein expression in RAW 264.7 cell in response to cigarette smoke extract. (**A**) Representative immunoblotting membranes of HDAC6, ICAM-1, and NOS2 in different experimental conditions. Quantification of (**B**) HDAC6/β-actin, (**C**) ICAM-1/β-actin, and (**D**) NOS2/β-actin protein expression in the different experimental conditions. Results are presented as mean ± SEM of three different experiments. * *p* <0.05, ** *p* < 0.01, *** *p* < 0.001, and **** *p* < 0.0001.

A Western blotting analysis was performed to investigate inflammatory pathways related to redox biology (Figure 9A). HDAC6 expression was significantly increased in the LPS (10 μg/mL) and CSE (5%) groups compared to the control, while HBx and KBx were effective in reducing it (Figure 9B). ICAM-1 expression was basal in the control group, while the LPS group (positive control; 10 μg/mL) displayed a modest upward trend. The CSE-only group (5%) showed a significant fourfold increase in ICAM-1 expression compared to the control, and treatment with HBx or KBx (200 μM) following CSE exposure reduced ICAM-1 expression (Figure 9C). NOS2 expression was significantly elevated in the CSE group compared to the control and the treatment with HBx and KBx (both at 200 μM) reduced it, keeping a similar expression to the control (Figure 9D).

### 2.8. Potassium Bixinate as Nrf2 Activator

**Figure 10:**
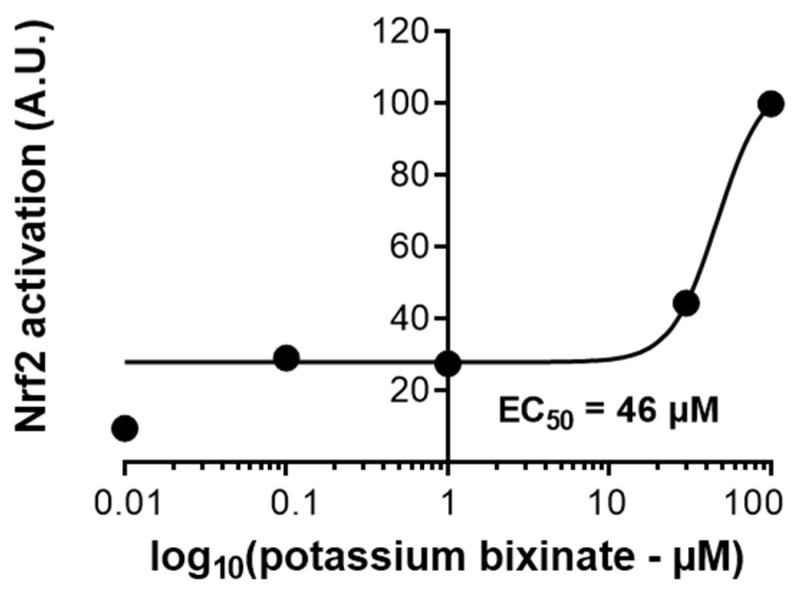
Potassium bixinate can activate the Nrf2/ARE axis with EC50 of 46 μM. Luciferase assay in RAW 264.7 lineage cells treated at different concentrations of KBx (0–200 μM).

The activation of transcription factor Nrf2 was evaluated using a luciferase assay with a plasmid containing the specific promoter region for the ARE sequence of DNA. RAW 264.7 cells transfected with this plasmid were exposed to different concentrations of KBx, and luciferase activity was measured in the sample supernatants as an indirect indicator of Nrf2 activation. A concentration–response curve was generated (Figure 10), and the EC50 for KBx in activating the Nrf2/ARE axis was calculated to be 46 μM.

### 2.9. Molecular Docking Revealed That Potassium Bixinate Interacts with Key Amino Acid Residues in Keap1 and HDAC6 Proteins

**Figure 11:**
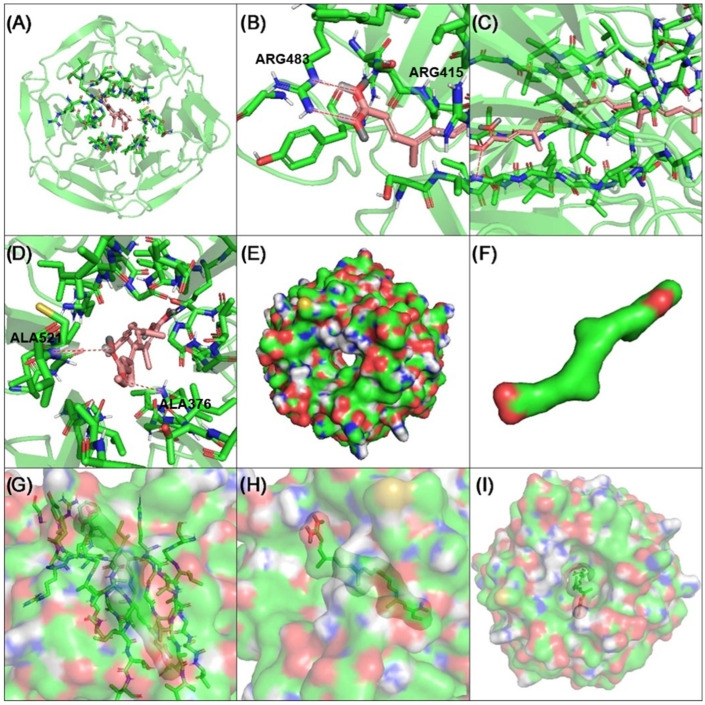
Molecular docking of bixinate with Keap1 (PDB: 4IFN). (**A**) Upper face of the Kelch domains of Keap1 docked with bixinate, (**B**) hydrogen bond between arginine 483 and carboxylate group, (**C**) interior of the pore of Keap1 accommodated with bixinate, (**D**) hydrogen bonds between the methyl ester of bixinate and peptide bonds of Keap1, polar surface of (**E**) Keap1 and (**F**) bixinate, (**G**) emphasis on the amino acids involved in the intermolecular interactions between Keap1 and bixinate, and (**H**,**I**) emphasis on bixinate and the polar areas of both.

A molecular docking analysis for Keap1 (Figure 11) was conducted to examine the mode of interaction between potassium bixinate and the protein (PDB: 4IFN). The methodology was validated by redocking the co-crystallized ligand presents in 4IFN using the ASP, ChemPLP, ChemScore, and GoldScore scoring functions. Supplementary Table S2 provides the RMSD values for each of the ten runs across the four scoring functions, along with their respective averages. The GoldScore function yielded the best RMSD value, closest to the resolution of the 4IFN crystal structure (2.159 vs. 2.40 Å), and was therefore used for the docking analysis of bixinate with Keap1. Figure 11 illustrates various aspects of the molecular interactions between the Kelch domains of the Keap1 protein and bixinate. Figure 11A displays the upper surface of the Keap1 Kelch domains bound to bixinate. In Figure 11B, the hydrogen bonds between arginine 483 and the carboxylate group of bixinate are highlighted. Figure 11C shows the interior of the Keap1 pore accommodating bixinate. In Figure 11D, the hydrogen bonds between the methyl ester group of bixinate and the peptide bonds of Keap1 are depicted. Figure 11E,F emphasizes the polar surfaces of Keap1 and bixinate, showcasing their interaction areas. Figure 11G focuses on the amino acids involved in the intermolecular interactions between Keap1 and bixinate. Finally, Figure 11H,I highlights bixinate and Keap1 and the polar regions of both. As described in the literature, arginines 415 and 483 are critical for the interaction between Keap1 and Nrf2 [23]. Therefore, it can be inferred that this interaction may be inhibited, leading to the release of Nrf2 from its suppressor protein, Keap1.

**Figure 12:**
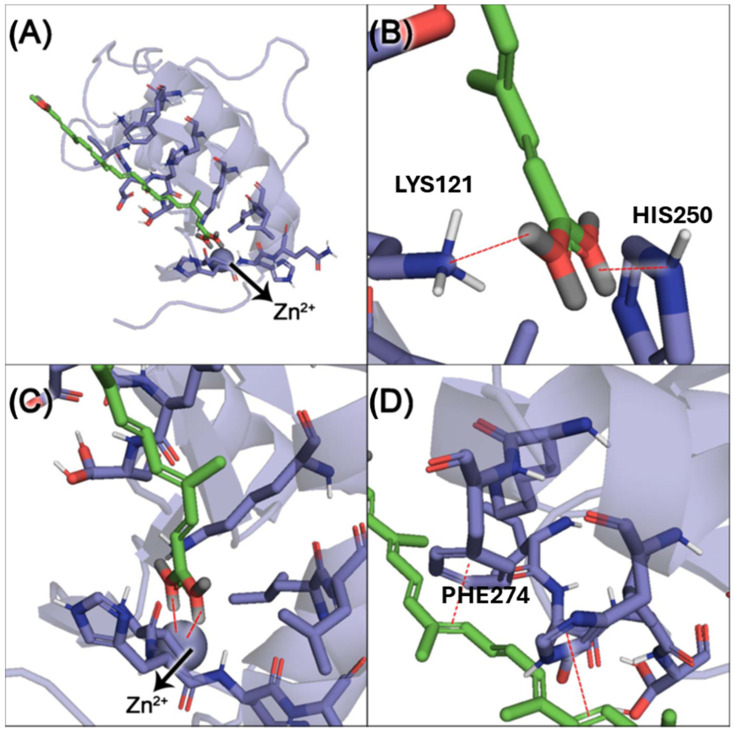
Molecular docking of bixinate with HDAC6 (PDB: 6UO2). (**A**,**C**) carboxylate group chelating the Zn^2+^ atom, (**B**) hydrogen bonds between K and H residues with carboxylate, and (**D**) dipole–dipole interactions between electrons π conjugated unsaturation and side-chains of aromatic amino acid residues (F and H).

The findings from the HDAC6 docking analysis are presented in Figure 12. This analysis aimed to investigate the interaction mode between potassium bixinate and the protein structure (PDB: 6UO2). To ensure methodological reliability, the co-crystallized ligand from 6UO2 was redocked using four scoring functions: ASP, ChemPLP, ChemScore, and GoldScore. Supplementary Table S3 details the RMSD values obtained for each of the ten runs across these scoring functions, along with their respective average values. ChemPLP scoring function was selected for the docking with KBx (1.00982 vs. 1.65 Å). As observed, the carboxylate group of KBx forms a hydrogen bond interaction with a histidine (H) and lysine (K) residue within the catalytic site (Figure 12B). Additionally, it chelates the Zn^2+^ atom (Figure 12A,C), while its π-electron-rich unsaturated hydrocarbon chain engages in dipole–dipole interactions with phenylalanine (F) and histidine (H) residues (Figure 12D).

### 2.10. UMAP of the COPD Cell Atlas for Nrf2 Gene Expression in Immune Cells

**Figure 13:**
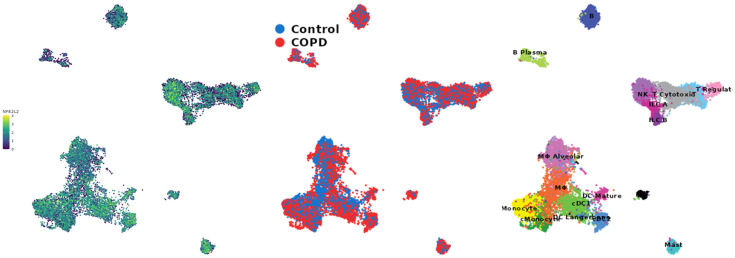
UMAP analysis of a COPD Cell Atlas, depicting Nrf2 gene expression across immune cells. The left panel shows Nrf2 expression levels (blue: low, yellow: high). The upper right panel identifies immune cell subpopulations (e.g., T lymphocytes, monocytes, dendritic cells, NK cells). The lower right panel compares cell distributions between control (blue) and COPD patients (red), highlighting a higher prevalence of cells with low Nrf2 expression in COPD, indicating potential antioxidant response impairment.

Figure 13 presents a UMAP analysis applied to a Chronic Obstructive Pulmonary Disease (COPD) Cell Atlas [24], highlighting the expression of the Nrf2 gene in different immune cells. In the left panel, the coloring indicates the expression levels of the *NFE2L2* (Nrf2) gene, with a color scale ranging from blue (low expression) to yellow (high expression). The upper right panel identifies immune cell subpopulations, while the lower right panel compares the distribution of cells from control individuals (blue) and COPD patients (red). The left panel of the image illustrates the variation in Nrf2 expression among different immune cells. Significant heterogeneity is observed, with cells exhibiting low (blue) to high (yellow) expression. Areas of higher cell density, indicative of widely represented cell populations, show moderate Nrf2 expression. In the upper right panel, various immune cell subpopulations have been identified, including T lymphocytes (CD4+ and CD8+), monocytes, dendritic cells, and natural killer (NK) cells, among others. The cellular diversity within the lung microenvironment is crucial for understanding the different inflammatory and antioxidant responses mediated by Nrf2. The lower right panel reveals the comparative distribution of cells between control individuals and COPD patients. The overlap of blue (control) and red (COPD) cells indicates significant phenotypic changes associated with the disease. In COPD patients, there is a greater representation of cells with low Nrf2 expression, suggesting a potential impairment in antioxidant response. This single-cell transcriptomic profiling reveals that Nrf2 expression within macrophages is highly heterogeneous, varying from low to high levels. This variability suggests that different macrophage subsets may have distinct capacities to regulate oxidative stress and inflammatory responses via the Nrf2 pathway. In macrophages, which play a pivotal role in COPD by producing inflammatory cytokines and ROS, the differential expression of Nrf2 indicates that certain subsets may be more effective in activating antioxidant responses, thereby reducing oxidative damage and inflammation. Conversely, macrophages with lower Nrf2 expression might contribute to the persistent inflammatory state observed in COPD.

### 2.11. Anti-Inflammatory Effects of Potassium Bixinate in Mice Challenged with LPS

**Figure 14:**
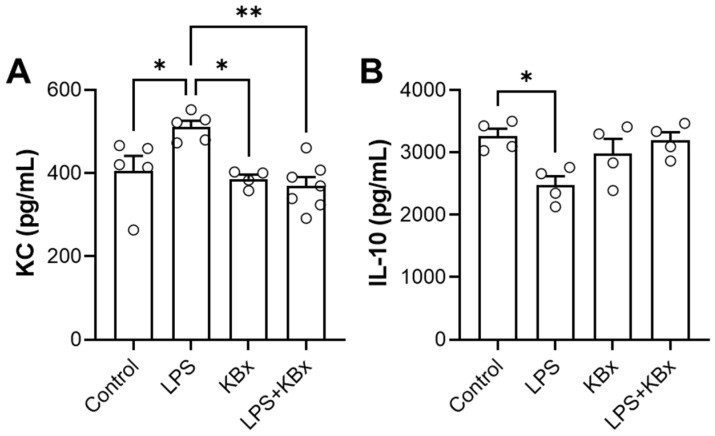
Potassium bixinate exhibits anti-inflammatory activity in vivo. ELISA for (**A**) KC and (**B**) IL-10 performed on the BAL of C57BL/6 mice challenged with intranasal LPS (60 μg) and pretreated with KBx (200 mg/kg). Results are presented as mean ± SEM of 4–7 animals. * *p* < 0.05 and ** *p* < 0.01.

BAL fluid from C57BL/6 mice challenged with intranasal LPS (i.n.—60 μg) and pretreated with KBx was analyzed to measure KC and IL-10 levels. KC levels were significantly higher in the LPS group compared to the control group (Figure 14A). KBx treatment alone had no significant effect on KC levels compared to the control. In the LPS + KBx group, KC levels were significantly lower compared to the LPS group, suggesting that KBx can attenuate the LPS-induced increase in KC. IL-10 levels were significantly lower in the LPS group compared to the control group (Figure 14B). The IL-10 levels in the KBx group were comparable to the control and similar to the LPS + KBx group, suggesting that KBx can increase IL-10 production in the presence of LPS. These results show that LPS treatment leads to an increase in KC and a decrease in IL-10, indicating an inflammatory response. KBx treatment appears to have anti-inflammatory effects, as it can reduce KC levels and increase IL-10 levels in the presence of LPS.

## 3. Discussion

This study aimed to synthesize potassium bixinate to improve its solubility and evaluate its potential to combat oxidative stress and inflammation caused by cigarette smoke and LPS. In vitro experiments on macrophages exposed to cigarette smoke extract assessed the antioxidant, anti-inflammatory, and anti-chemotactic properties of potassium bixinate. In vivo studies on mice evaluated its anti-inflammatory effects. Mechanistic studies, including molecular docking, investigated how bixinate activates the Nrf2 pathway and interacts with HDAC6, providing insights into its potential biological activity and mechanisms of action.

Bixin, a natural pigment, typically exists in the more stable E (all-trans) form [25]. However, this study detected the presence of the less common Z (all-cis) isomer. This finding suggests potential differences in processing conditions or environmental factors that favored the formation of the Z isomer. The Z isomer may possess unique physicochemical properties and biological properties. All subsequent experiments in this study were conducted exclusively with the Z-bixin isomer, marking the first reported use of Z-bixin in biological assays.

This study shows that HBx has limited solubility in acidic conditions (pH 2.0), but its solubility improves at neutral to slightly basic pH levels (6.6 and 7.4). This suggests that bixin is more likely to be absorbed in the upper small intestine than in the stomach [26]. However, its solubility decreases over 24 h at pH 7.4, indicating potential precipitation and reduced bioavailability. The low permeability and absorbed fraction suggest limited oral bioavailability for HBx. Enhancements or alternative delivery methods may be needed to improve absorption. Despite these challenges, bixin shows high plasmatic and microsomal hydrolysis stability, supporting its potential as a therapeutic agent. To improve solubility and bioavailability, the synthesis of bixin’s potassium salt (KBx) is a promising approach, as salt formation typically enhances solubility and absorption [19,27]. KBx could provide better pharmacokinetic properties, overcoming HBx’s limitations and improving its oral delivery and therapeutic efficacy. This study aimed to produce bixin potassium salt and assess its ability to retain HBx’s anti-inflammatory and antioxidant properties, with in vitro and in vivo models using RAW 264.7 cells and intranasal LPS administration. The synthesis of the KBx was performed using the well-established solvent evaporation method [19,20]. Solubility tests confirmed a significant increase in the aqueous solubility of the product, and carotenoid tests indicated no degradation of bixin during synthesis. Mass spectrometry revealed peaks corresponding to bixinate (395.2220 *m*/*z*) and its potassium adduct (433.1778 *m*/*z*), confirming the expected formation of KBx without evidence of bixin hydrolysis. Additionally, the absence of the broadband corresponding to a hydroxyl group in the KBx FTIR further supports the successful salt formation [28]. The melting point of KBx was approximately 10 °C lower than HBx (187–191 °C vs. 199–204 °C), enhancing its solubility due to reduced intermolecular interactions [29,30,31]. TEM images showed KBx as an amorphous solid with irregular edges and little crystalline organization, a form known to improve solubility by reducing the energetic barrier for molecular solvation. These findings support the successful synthesis of KBx with properties that enhance its potential for improved solubility and therapeutic application.

Bixin shows promise in modulating oxidative stress and inflammation, with Nrf2 activation being a key mechanism. Both HBx and its potassium derivative (KBx) demonstrated no cytotoxicity in vitro, maintaining cell viability up to 200 µM. At this concentration, both compounds effectively reduced ROS formation, supporting its selection for further experiments. Cells exposed to CSE showed decreased viability at 5% concentration, which was used in subsequent studies due to the high content of toxic components like nicotine, PAHs, and electrophilic substances [32].

CSE exposure increased ICAM-1 expression, contributing to immune cell recruitment and inflammation. However, treatment with HBx or KBx reversed ICAM-1 expression to baseline levels, reducing cell migration and macrophage presence in the BAL [33], corroborating our previous study with polymeric nanoparticles of bixin, although this mechanism was not investigated on this occasion [2]. This effect is likely mediated by HBx and KBx, which modulate ICAM-1 and reduce NF-κB activation by limiting p-p65 nuclear translocation [34,35,36,37]. Additionally, both compounds inhibited actin filament polymerization, crucial for cell migration, further reducing migratory capacity in the experimental model. HDAC6, a key enzyme in actin polymerization during inflammation [38,39], may also play a role in this process, since molecular docking shows bixinate to chelate the zinc atom in catalytic site of the enzyme. The production of ROS and reactive nitrogen species (RNS) during inflammation is closely linked to proteins such as NOS2 (inducible nitric oxide synthase) [17], which marks the M1 pro-inflammatory macrophage profile. Elevated NOS2 expression and activity lead to excessive nitric oxide (NO) production [40]. While physiological levels of NO support tissue homeostasis, excessive NO reacts with superoxide to form peroxynitrite, causing nitrative protein damage [41]. In cells exposed to CSE, NOS2 levels were increased, but it was reversed by HBx and KBx, demonstrating their ability to prevent nitrative protein damage.

Nicotine, a key CSE component, activates the NF-κB pathway, leading to the nuclear translocation of the p65 subunit and the transcription of pro-inflammatory genes like KC, TNF-α, NOS2, and ICAM-1 [34,42]. CSE exposure heightened nuclear p-p65 levels, which were reduced by HBx and KBx, highlighting their role in inhibiting this pro-inflammatory pathway. To confirm KBx’s antioxidant capacity, a luciferase assay showed that it maintained Nrf2/ARE pathway activation and scavenged ROS effectively. Molecular docking analysis revealed that bixinate interacts with Keap1 through a protein–protein interaction inhibition mechanism rather than classical electrophilic activation, effectively inhibiting Nrf2-Keap1 interaction [23]. These findings underscore HBx and KBx’s potential to modulate oxidative stress and inflammation via Nrf2 and NF-κB pathways.

The in vivo experiment demonstrated that KBx, the potassium salt derivative of bixin, is absorbed via the gastrointestinal tract and preserves its anti-inflammatory properties. Mice pretreated with KBx showed reduced KC levels and increased IL-10 in BAL fluid after LPS-induced lung inflammation, confirming KBx’s therapeutic potential. Bixin and KBx also reduced markers of inflammation such as TNF-α, IL-6, and neutrophil influx in lung tissue, primarily through Nrf2 activation, which prevents its degradation and promotes nuclear translocation, reducing ROS levels and oxidative stress.

Nrf2 plays a critical role in modulating immune response in COPD, with reduced expression linked to chronic inflammation and oxidative stress [41,42]. RAW 264.7 macrophages were used as a model to study Nrf2 activation due to their relevance in inflammatory and oxidative stress responses, including their polarization to an M2 phenotype that aids in inflammation resolution. Single-cell analyses from the COPD Cell Atlas revealed heterogeneous Nrf2 expression across immune cell types, emphasizing its potential as a therapeutic target [24]. The Nrf2/ARE signaling pathway is essential for defense against oxidative stress, and bixin has been shown to modulate this pathway by releasing Nrf2 from Keap1, allowing for antioxidant gene activation.

Previous studies in mice using bixin-loaded nanoparticles showed promising anti-inflammatory effects [2,7], but the compound’s low solubility limited its application. The development of KBx addresses this limitation by enhancing solubility and enabling effectiveness in vivo absorption without nanotechnology, making long-term treatments more feasible and cost-effective. These findings underscore the therapeutic promise of HBx and KBx for chronic inflammatory diseases like COPD. Future research should focus on optimizing dosages, ensuring long-term safety, and further elucidating the mechanisms of Nrf2 activation and its effects on immune cell function and inflammation.

In conclusion, this study is the first to report the use of the Z-isomer of HBx in both in vivo and in vitro experiments, as well as the development of its potassium salt derivative for treatment purposes. This approach successfully addressed the challenges associated with HBx’s chemical structure that hinder its oral absorption, making it a viable candidate for therapeutic use. KBx demonstrated bioavailability and effectiveness while retaining the anti-inflammatory and antioxidant properties previously described for HBx, offering potential as a treatment targeting macrophage and lung tissue stressors.

## 4. Materials and Methods

### 4.1. Reagents

Annatto’s seeds were purchased from Florien Fitoativos (São Paulo, SP, Brazil). Hexane, chloroform, methanol, ethanol, isopropyl alcohol, dodecane, dimethyl sulfoxide (DMSO), acetonitrile for HPLC, methanol for HPLC, pH 2.0 buffered solution, L-α-soy phosphatidylcholine, acyclovir, ceftriaxone, coumarin, ofloxacin, verapamil, nitro-tetrazolium blue chloride (NBT), lipopolysaccharide (LPS), SIGMAFAST™ Protease Inhibitor, sodium orthovanadate, bovine serum albumin (BSA), mitomycin C, RIPA buffer, ethylenediaminetetraacetic acid (EDTA), methylthiazolyldiphenyl-tetrazolium bromide (MTT), hydrochloric acid (HCl), sodium hydroxide (NaOH), synthetic poly-vinylidene fluoride (PVDF) membrane, potassium hydroxide (KOH), sodium chloride (NaCl), sodium dihydrogen phosphate (NaH_2_PO_4_), sodium hydrogen phosphate (Na_2_HPO_4_), potassium phosphate dibasic (K_2_HPO_4_), potassium phosphate monobasic (KH_2_PO_4_), sodium formate, Tween-20, and heparin were obtained from Sigma-Aldrich (Saint Louis, MO, USA). ActinRed, DAPI (4′,6-diamidino-2-phenylindole), SuperSignal West Femto Maximum Sensitivity Substrate is an ultra-sensitive enhanced chemiluminescent (ECL) substrate, BCA Protein Assay Kit, Opti-MEM, phosphate-buffered saline (PBS) at pH 7.4, Dulbecco’s Modified Eagle Medium (DMEM), fetal bovine serum (FBS), DMEM FluoroBrite, and Lipofectamine 2000 were obtained from ThermoFisher Scientific (Waltham, MA, USA). DMSO-d6 containing 0.05% *v*/*v* TMS was obtained from Cambridge Isotope Laboratories, Inc. (Cambridge, MA, USA). Immun-Blot^®^ PVDF membrane, glycine, Tris-HCl, sodium dodecyl sulfate (SDS), 10× Tris/Glycine/SDS buffer, 10× Tris/Glycine buffer, and 30% Acrylamide/Bis Solution (37.5:1) were obtained from Bio-Rad Laboratórios do Brasil (São Paulo, Brazil). Isoflurane was obtained from Cristália Produtos Químicos e Farmacêuticos (Rio de Janeiro, Brazil).

### 4.2. Bixin Purification, Characterization, and Salt Synthesis

The annatto’s seeds underwent a two-step Soxhlet extraction process. First, hexane was used for 8 h, followed by chloroform extraction for another 8 h. The red organic phase obtained from the chloroform extraction was concentrated using a rotary evaporator. The remaining residue was then dissolved in a 5% (*w*/*v*) methanolic NaOH solution under magnetic stirring for 45 min. The resulting alkaline solution was filtered to remove insoluble impurities. Bixin was precipitated by adding diluted HCl solution until the pH reached 4. The precipitate was then filtered using a vacuum apparatus. The solid material retained on the filter was washed with distilled water until a neutral pH was achieved. Finally, it was dried at 70 °C until all residual solvents evaporated completely [2,7].

Bixin purification was achieved by recrystallizing a hot 9:1 ethanol:water mixture. The extracted powder was dissolved in hot ethanol under magnetic stirring, filtered, and mixed with hot water in the appropriate ratio. The solution was left at 25 °C for 24 h to allow for bixin recrystallization. Analytical HPLC was used to determine the compound’s purity. The HPLC system employed an LC-20AD Shimadzu (Kyoto, Japan) equipped with a 100-5C18 Kromasil (Bohus, Sweden) analytical column (4.6 mm × 250 mm, 5 μm). Detection was performed with a SPD-M20A Shimadzu (Kyoto, Japan) detector set at 254 nm and 457 nm wavelengths. The isocratic mobile phase for HPLC analysis consisted of 85% acetonitrile and 15% methanol at 22 °C with a 1 mL/min flow rate.

Bixin was characterized using an externally calibrated electrospray ionization spectrometer, amaZon SL model Esquire 6000—ESI Ion Trap Msn System Bruker (Billerica, MA, USA). Sodium formate was used for external calibration in a range from 75 to 1200 *m*/*z*. Infrared (IR) spectra (cm^−1^) were obtained using a Nicolet iS10 FTIR Spectrometer (ThermoFisher Scientific, Waltham, MA, USA) in a range from 400 to 4000 cm^−1^. The melting point of bixin was determined using a manual fusiometer Q340.23 Quimis (São Paulo, Brazil) with a glass capillary method.

Nuclear magnetic resonance (NMR) analyses were performed on a 2.0 mg sample of bixin dissolved in 600 μL of DMSO-d6 containing 0.05% *v*/*v* TMS. All spectra were acquired on an Agilent VNMRS-500 spectrometer (^1^H at 499.79 MHz and ^13^C at 125.68 MHz) (Santa Clara, CA, USA) at 25 °C and processed using MestReNova software v. 14.3.3. The following parameters were used for spectral acquisition:

- ^1^H NMR (64 scans);
- ^1^H–^13^C HSQC pure shift (16 scans, 1500 points in f2 and 400 in f1);
- ^1^H–^13^C HMBC (32 scans, 901 points in f2 and 512 in f1);
- ^1^H–^1^H COSY (16 scans, 901 points in f2 and 512 in f1);
- ^1^H–^1^H NOESY (200 ms mixing time, 16 scans, 901 points in f2 and 512 in f1).

Potassium bixinate was prepared using a solvent evaporation method. The reaction involved bixin and KOH crystals in a 1:1 stoichiometric ratio within an aqueous medium. The reaction was carried out in a round-bottomed flask in an ice bath. After complete solubilization of the bixin, the solution was transferred to an oil bath for complete solvent evaporation, resulting in a potassium bixinate precipitate. Potassium bixinate was characterized using the same methods employed for bixin, including thermal analysis (melting point), physical analysis (MS), and physicochemical analysis (IR). For transmission electron microscopy (TEM) analysis, 10 μL of a 1 mg/mL solution was dropped onto a 300-mesh copper grid without staining, and images were obtained using a HT7800 HITACHI (Tokyo, Japan) microscope at 100 kV.

#### 4.2.1. Bixin

^1^H NMR (500 MHz, DMSO-d6): δ 7.89 (d, *J* = 15.4 Hz, 1H), 7.27 (d, *J* = 15.5 Hz, 1H), 6.93–6.83 (m, 1H), 6.84–6.78 (m, 2H), 6.71 (dd, *J* = 14.7, 11.2 Hz, 1H), 6.62 (t, *J* = 14.2 Hz, 2H), 6.57–6.42 (m, 4H), 5.95 (d, *J* = 15.4 Hz, 1H), 5.83 (d, *J* = 15.5 Hz, 1H), 3.70 (s, 3H), 1.96 (dd, 12H). ^13^C NMR, (DMSO-d_6_, 125 MHz): δ 167.66 (C1), 148.05 (C3), 141.31 (C7), 140.43 (C14), 139.75 (C18), 138.87 (C5), 137.89 (C16), 136.62 (C8), 134.38 (C12), 133.22 (C4), 131.25 (C11), 130.99 (C17), 124.61 (C6), 122.95 (C15), 117.48 (C19), 116.99 (C2), 51.27 (C20), 19.73 (C17′), 12.50 (C13′), 12.30 (C4′). HPLC_457nm_: 97.78% Melting point: 199–204 °C. MS: 395.22 *m*/*z*. FTIR: 727 cm^−1^, 964 cm^−1^, 1159 cm^−1^, 1379 cm^−1^, 1431 cm^−1^, 1608 cm^−1^, 1717 cm^−1^, 2853 cm^−1^, 2922 cm^−1^ and 3426 cm^−1^.

#### 4.2.2. Potassium Bixinate

HPLC_457nm_: 95.56% Melting point: 187–191 °C. MS: 433.1778 *m*/*z*. FTIR: 1172 cm^−1^, 1421 cm^−1^, 1557 cm^−1^, 1692 cm^−1^ and 2851 cm^−1^.

### 4.3. Drug-like Properties Determination

For the kinetic solubility assay, a 10 mM stock solution of bixin was analytically prepared in DMSO and then diluted in different buffered solutions at pH 2.0 (commercial), 6.8 (potassium-phosphate-buffered saline solution (KPE) buffer—100 mM K_2_HPO_4_, 100 mM KH_2_PO_4_ and 5 mM EDTA), and 7.4 (PBS buffer) to a final concentration of 200 μM. These solutions were incubated in a shaking water bath at 37 °C for 4 and 24 h. Immediately after each incubation period, the samples were centrifuged at 21,000× *g* Universal Centrifuge 320R Hettich (Tuttlingen, Germany) at 22 °C. The supernatant was collected, filtered through a 0.44 μm Millipore filter (Sigma-Aldrich—Saint Louis, MO, USA) into a vial, and analyzed using a HPLC LC-20AD Shimadzu (Kyoto, Japan) coupled with a DAD detector set at 457 nm. The mobile phase consisted of acetonitrile and methanol (85:15 *v*/*v*) using a 100-5C18 Kromasil (Bohus, Sweden) analytical column (4.6 mm × 250 mm, 5 μm) as the stationary phase in isocratic mode at 22 °C with a flow rate of 1 mL/min. A calibration curve was prepared using bixin in acetonitrile at concentrations ranging from 0 to 200 μM and analyzed under the same HPLC conditions. LogS was calculated as the base-10 logarithm of the solubility (in μM) for each time point.

The parallel artificial membrane permeability assay (PAMPA) utilizes a 96-well plate system in a “sandwich” format, where one plate is positioned above another (PAMPA) [43]. The upper plate, known as the donor compartment, contains the compounds (test or control) dissolved in a buffered solution. This compartment features a PVDF membrane impregnated with a lipid solution, creating a barrier that allows for the compounds to diffuse to the lower plate, termed the receptor compartment [44]. The lipid composition of the PVDF filter varies depending on the type of permeability test. For gastrointestinal tract (GIT) permeability assessments, L-α-soy phosphatidylcholine in dodecane is used. Optical density values are measured at specified wavelengths for each compound and compared against controls. For GIT permeability, the absorbed fraction (Fa%) and permeability (Pe) are determined. PAMPA-GIT results categorize compounds based on their absorbed fraction as having high (70–100%), medium (30–69%), or low (0–29%) intestinal permeability. Compounds for these assays were diluted from a 10 mM stock solution. These assays were conducted using a 1 mg/mL stock solution per compound. The PAMPA’s validity was confirmed by comparing experimental data with the literature reports on the artificial membrane permeability of various drugs (acyclovir, ceftriaxone, coumarin, norfloxacin, and verapamil) [22].

### 4.4. Rat Plasma and Microsomal Stability

Two- to three-month-old healthy male Wistar rats (with an average weight of 385 g) were obtained from the Graduation Program in Pharmacology and Medicinal Chemistry Bioterium (CCS/UFRJ) (Rio de Janeiro, Brazil). The rats were fed ad libitum with Purina chow (Nuvilab, Curitiba, Brazil) and had unrestricted access to water. They were housed in a controlled environment maintained at 22 ± 2 °C, 50–70% relative humidity and a 12 h light/dark cycle. The local Animal Ethics Committee approved all experimental procedures (046/21).

A 10 mM stock solution of bixin was prepared in DMSO. Rat blood was collected with heparin and centrifuged. The plasma fraction was separated and diluted to 80% in PBS at pH 7.4. Then, 50 μL of the 80% rat plasma solution was added to a final volume of 250 μL of a KPE at pH 7.4 containing 50 μM of bixin. The bixin was incubated with rat plasma under these conditions for 0, 30, 60, 120, and 240 min with shaking in a water bath at 37 °C. Immediately after the incubation time, proteins were precipitated, and bixin was extracted by adding 1000 μL of cold acetonitrile: methanol (1:1) mixture containing 5 μM of an internal standard. The resulting solution was centrifuged at 20,000× *g* in a Universal Centrifuge 320R Hettich (Tuttlingen, Germany) at 4 °C for 15 min. The supernatant was collected, filtered through a 0.22 μm filter into a vial, and then analyzed by HPLC using an LC-20AD Shimadzu (Kyoto, Japan) system equipped with a 100-5C18 Kromasil (Bohus, Sweden) analytical column (4.6 mm × 250 mm, 5 μm) and an SPD M20A diode array detector Shimadzu (Kyoto, Japan) set to wavelengths of 271 nm and 457 nm. The solvent system for the HPLC analysis consisted of acetonitrile and methanol in an 85:15 ratio in isocratic mode at 22 °C with a flow rate of 1 mL/min.

A final solution of microsomes (1 mg/mL protein) in 250 μL of PBS at pH 7.4 containing 50 μM of bixin was prepared in the absence of an NADPH-regenerating metabolic cofactor system. The bixin was incubated with rat microsomes under these conditions for 0, 30, 60, 120, and 240 min with shaking in a water bath at 37 °C. The subsequent steps for protein precipitation, bixin extraction, and HPLC analysis were identical to those described for the rat plasma stability assay.

### 4.5. Cigarette Smoke Extract (CSE) Preparation

CSE was prepared immediately before each experiment. The extract was obtained by collecting the smoke from a burning cigarette (Marlboro^®^, 10 mg tar, 0.8 mg nicotine, and 10 mg carbon monoxide—Richmond, VA, USA) and transferring it into 10 mL of DMEM at a rate of 1/2 cigarette per minute. The pH was adjusted to 7.4, when necessary, immediately after the procedure, and the resulting solution was filtered using a 0.22 μm Millipore filter (Sigma-Aldrich—Saint Louis, MO, USA). The final concentration of this solution was expressed as a percentage (100% corresponds to 1 cigarette per 1 mL of medium). A serial dilution of the CSE was performed to achieve concentrations of 0.65%, 1.25%, 2.5%, 5%, and 10%. CSE quality control was conducted by measuring the absorbance at 320 nm.

### 4.6. RAW 264.7 Macrophage Cell Line and Experimental Design

The RAW 264.7 macrophage cell lines were cultured in DMEM supplemented with 10% (*v*/*v*) FBS, pH 7.2, at 37 °C in a humidified atmosphere with 5% CO_2_. RAW 264.7 cells were plated in a 24-well plate (3.5 × 10^5^ cells/well) in DMEM with 1% (*v*/*v*) FBS and left overnight to adhere. Subsequently, 5% CSE in DMEM was added to each well for 2 h. After incubation, the medium was removed, and a 100 μM solution of bixin or potassium bixinate (diluted in DMEM; bixin was first prepared as a stock solution in DMSO) was added for an additional 1 h.

### 4.7. MTT Assay

RAW 264.7 cells were plated (5 × 10^4^ cells/well) in a 96-well plate with DMEM and 10% FBS. When cells reached 90% confluence, the 10% (*v*/*v*) FBS DMEM was discarded and replaced with the stimuli. The plate was incubated at 37 °C and 5% CO_2_ for 4 h, after which MTT solution (5 mg/mL) in DMEM 1% (*v*/*v*) FBS was added. The metabolism of MTT by the cells was monitored by the formation of insoluble salts, which were then dissolved in isopropyl alcohol. Absorbance was read at 570 nm using a Varioskan LUX (ThermoFisher Scientific, Waltham, MA, USA). Cell viability was assessed at different concentrations of CSE (0–10%), bixin, and potassium bixinate (0–200 μM). Cell viability was normalized in all groups by the control (1% (*v*/*v*) FBS DMEM), with 10% (*v*/*v*) FBS in DMEM used as the positive growth control.

### 4.8. ROS Scavenger Activity Assay

ROS levels were determined using the NBT method [45]. RAW 264.7 cells were incubated for 1 h with 10 μg/mL LPS and different concentrations of bixin or potassium bixinate (0–200 μM) to evaluate their ability to intercept superoxide anions induced by LPS. After incubation, a 10% (*w*/*v*) NBT solution was added to each well and incubated for 1 h at 37 °C. Cellular membranes were permeabilized with 2 M KOH, and the resulting formazan was solubilized in DMSO. Optical density (OD) was measured at 630 nm using a Varioskan LUX spectrophotometer (ThermoFisher Scientific, Waltham, MA, USA).

### 4.9. Cell Lysate, Protein Content Determination, and Western Blotting

Cells were lysed in RIPA buffer containing SIGMAFAST™ Protease Inhibitor and 1 mM sodium orthovanadate. Samples were then analyzed using the bicinchoninic acid (BCA) method (BCA Protein Assay Kit). Standard curves with known albumin concentrations (0–2 mg/mL) were used. The working solution was prepared according to the manufacturer’s instructions. For protein quantification, 10 μL of the sample was mixed with 100 μL of the working solution, and the same procedure was performed for each point on the standard curve in a 96-well plate. The plate was incubated at 37 °C, protected from light, to allow for the reaction to occur and the colorimetric complex to form. Absorbance was read at 565 nm using a Varioskan LUX spectrophotometer (ThermoFisher Scientific, Waltham, MA, USA).

Cell lysates (10–20 μg of protein) were separated by SDS-PAGE (sodium dodecyl sulfate polyacrylamide gel for electrophoresis) (8 or 12% polyacrylamide) and transferred (25 V, 1.0 A, 30 min—Trans-Blot Turbo Transfer System, Bio-Rad—São Paulo, Brazil) to a PVDF membrane. The proteins were incubated overnight at 4 °C with the following primary antibodies: anti-ICAM-1 (gt5811-goat-1:250; gel of 10%) (Sigma-Aldrich, Saint Louis, MO, USA), anti-NOS2 (ab178945-rabbit-1:1000; gel of 8%) (Abcam—Cambridge, United Kingdom), anti-HDAC6 (#7612-rabbit-1:1000; gel of 8%) (Cell Signaling Technology—Danvers, MA, USA), and anti-β-actin (a5441-mouse-1:5000; gel of 12%) (Sigma-Aldrich—Saint Louis, MO, USA). Membranes were then incubated for at least 1 h with peroxidase-conjugated secondary antibodies (ab6789-mouse-1:10,000, ab6721-rabbit-1:10,000, or ab6741-goat-1:10,000) (Abcam, Cambridge, UK). Immunoreactive proteins were visualized using chemiluminescence with the assistance of an ECL solution, and membranes were developed and photographed using a ImageQuant™ LAS500, LAS4000 (GE Healthcare, Chicago, IL, USA) or a ChemiDoc (Bio-Rad, São Paulo, Brazil). Densitometry was quantified using ImageJ software v. 1.54p. Results are expressed as densitometric values for each protein normalized to β-actin.

### 4.10. Wound Healing Migration Assay

RAW 264.7 cells were seeded in a 24-well plate (4.0 × 10^5^ cells/well) and cultured until maximum confluence. Cells were then treated with mitomycin C (5 μg/mL) in FBS-free DMEM for 2 h, followed by washing with PBS. A linear scratch was made with a 200 μL pipette tip in each well. Cells were then stimulated for 24 h with the stimuli diluted in DMEM supplemented with 1% (*v*/*v*) FBS. Wound closure was monitored by capturing images at 0 and 24 h. The area of wound closure in each image was measured at five different points using a ruler tool, and an average was calculated for each image and condition. Cell migration was quantified as the percentage of wound closure at 24 h compared to the area at 0 h for each group.

### 4.11. Immunocytochemistry for p-p65

RAW 264.7 cells were seeded in a 24-well plate (2.5 × 10^5^ cells/well) and stimulated as previously described. After reaching 70% confluence, cells were blocked with 5% (*w*/*v*) BSA for 1 h, then washed three times in PBS-Tween 20 (0.1% (*v*/*v*)). The supernatants were replaced with a solution containing the primary antibody against p-p65 (#3033-rabbit-1:500) (Cell Signaling Technology, Danvers, MA, USA) diluted in PBS-T with 1% (*w*/*v*) BSA and incubated overnight. After incubation, cells were incubated for 1 h with a secondary antibody conjugated to Alexa Fluor-488 (1:1000; ThermoFisher Scientific, Waltham, MA, USA). Cells were then washed three times with PBS-T and incubated with DAPI for 5 min. After the final wash, DMEM FluoroBrite was added, and fluorescence was analyzed using the EVOS inverted microscope (ThermoFisher Scientific, Waltham, MA, USA). Images were captured at 20× magnification from at least 10 fields for each experimental condition, and fluorescence intensity was quantified.

### 4.12. F-Actin Polymerization Assay

RAW 264.7 cells were seeded in a 24-well plate (2.5 × 10^5^ cells/well) and stimulated as previously described. After reaching 70% confluence, cells were blocked with 5% (*w*/*v*) BSA for 1 h and washed three times in PBS-Tween 20 (0.1% (*v*/*v*)). The supernatants were replaced with ActinRed (ThermoFisher Scientific, Waltham, MA, USA) for 30 min, followed by washing and incubation with DAPI for 5 min. After the final wash, DMEM FluoroBrite was added, and fluorescence was analyzed using the EVOS inverted microscope (ThermoFisher Scientific, Waltham, MA, USA).

### 4.13. Luciferase ARE (Antioxidant Response Element) Reporter Assay

RAW 264.7 cells (3.5 × 10^5^ cells/well) were seeded into 24-well plates in FBS-free DMEM. Next day, cells were transfected with the ARE-responsive luciferase reporter kit (#60514, BPS Bioscience, San Diego, CA, USA) in Opti-MEM containing Lipofectamine 2000 and incubated for 24 h. After stimulation with different concentrations of potassium bixinate (0–200 μM), the luciferase-containing medium was collected and incubated with luciferin substrate (Dual Luciferase Assay System) (#60683-1, BPS Bioscience, San Diego, CA, USA) according to the manufacturer’s instructions. Luminescence emitted by luciferin cleavage was measured using the Varioskan LUX plate reader (ThermoFisher Scientific, Waltham, MA, USA).

### 4.14. Mouse Experimental Design

Eight-week-old male C57BL/6 mice were obtained from the Multidisciplinary Center for Biological Research in Laboratory Animal Science (CEMIB/UNICAMP, Campinas, Brazil). The mice were provided with Purina chow ad libitum (Nuvilab, Curitiba, Brazil) and had unrestricted access to water in a controlled environment maintained at 22 ± 2 °C, 50–70% relative humidity, and a 12 h light/dark cycle (EB-273B, Insight Equipamentos Ltd., São Paulo, Brazil). They were acclimatized for two weeks before the experimental procedures. All experimental protocols were approved by the local Animal Ethics Committee (096/19).

To evaluate the pharmacological effects of potassium bixinate, the animals were divided into four groups (n = 5–7): (1) Control, (2) KBx, (3) LPS, and (4) LPS + KBx. For five consecutive days, animals in groups 2 and 4 were treated by oral gavage with 200 mg/kg of potassium bixinate diluted in PBS in a final volume of 100 μL, while animals in groups 1 and 3 were treated with 100 μL of PBS. On the sixth day, animals in groups 3 and 4 were challenged intranasally with 60 μg of LPS (extracted from *Escherichia coli* O55:B5) diluted in PBS (10 μL), while animals in groups 1 and 2 received 10 μL of PBS intranasally. Sixteen hours after LPS/PBS instillation, the animals were euthanized by cervical dislocation under isoflurane sedation.

### 4.15. Bronchoalveolar Lavage (BAL) Collection

Immediately following euthanasia, BAL was collected using an intratracheal cannula. The lavage was performed with 1.5 mL of saline solution (3 × 0.5 mL). The collected lavage fluid was centrifuged at 600× *g* for 10 min, and the supernatant was stored at −80 °C for subsequent enzyme-linked immunosorbent assay (ELISA) analysis.

### 4.16. ELISA for BAL Cytokine Measurement

Cytokine levels (IL-10 and KC; #900-K53K and #900-K127K) were quantified in BAL samples using an ELISA kit, following the manufacturer’s instructions (PeproTech, Neuilly Sur-Seine, France). A sample volume of 25 μL was used without dilution. The enzymatic reaction was initiated with peroxidase and a specific substrate, and the reaction was stopped before measuring absorbance at 450 nm and 570 nm using a Varioskan LUX spectrometer (ThermoFisher Scientific, Waltham, MA, USA). A calibration curve was generated using standard concentrations of the target protein, with the method showing linearity across a range of 2000–0 pg/mL.

### 4.17. In Silico Study

Molecular docking simulations were conducted using the Genetic Optimization for Ligand Docking (GOLD) software v. 5.6. The crystallographic structure of Keap1, identified by the PDB code 4IFN (resolution 2.40 Å), was obtained from the Protein Data Bank (PDB; http://www.rcsb.org, accessed on 13 March 2025). The co-crystallized ligand associated with this structure served as a reference for validating the docking methodology and defining the binding site. Hydrogen atoms were added to the protein, and the binding pocket was delineated using the co-crystallized ligand and all amino acid residues within a 6 Å radius. Redocking of the co-crystallized ligand was performed to assess the reliability of the docking protocol. The validation of the methodology was determined by calculating the root-mean-square deviation (RMSD) between the experimentally observed pose and the redocked pose. The RMSD values obtained using the ASP (6.687), ChemPLP (5.938), ChemScore (6.922), and GoldScore (2.159) scoring functions were analyzed, and the scoring function with the best performance was selected for subsequent docking studies.

The structure of potassium bixinate was constructed, and its protonation state was determined using the Percepta software v. 2012. The molecule’s geometry was then optimized using the PM6 (Parametric Method 6) semi-empirical approach to generate the lowest-energy conformer for use in docking studies. Additionally, another docking was performed within the bixinate and HDAC6 crystal structure (PDB: 6UO2—resolution: 1.65 Å), following the same parameters used for the 4IFN. The redocking scoring functions results for validation were as follows: ASP (RMSD: 1.05684), ChemPLP (RMSD: 1.00982), ChemScore (RMSD: 1.88182), and GoldScore (RMSD: 1.08718).

### 4.18. COPD Cell Atlas Single-Cell Gene Expression Analysis

A search for the NFE2L2 gene was conducted in the COPD Cell Atlas database (http://www.copdcellatlas.com, accessed on 23 April 2024) [24] using the UMAP (Uniform Manifold Approximation and Projection) model across all cell subtype categories. An empirical analysis of its gene expression distribution was performed.

### 4.19. Statistical Analysis

All experiments were performed in triplicate, and the results are expressed as the mean ± SEM (Standard Error of the Mean). Statistical analyses were conducted using one-way ANOVA followed by Bonferroni’s post hoc test. GraphPad Prism software v. 8.0 was used for all statistical calculations. Statistical significance was set at *p* < 0.05.

## 5. Conclusions

This study develops a potassium salt of bixin, addressing the challenges associated with its chemical structure derived from carotenoids. This approach allows for its potential use as a treatment against stressors in macrophages and lung tissue. Potassium bixinate was found to be bioavailable and effective, maintaining the anti-inflammatory and antioxidant properties previously described for its precursor, bixin. Additionally, a potential new mechanism of action through iHDAC6 activity, reducing cell motility, was observed, a feature not previously described for bixin.

## Figures and Tables

**Table 1:** Permeability coefficient of standard drugs, used as control, and HBx in the PAMPA-TGI assay.

Compound	Literature Pe (10^−6^ cm/s)	Experimental Pe (10^−6^ cm/s)	Literature Fa (%)	Experimental Fa (%)	Classification
Acyclovir	0.06	0.36	21	12.64	Low
Ceftriaxone	0.1	0.53	1	18.19	Low
Norfloxacin	0.9	1.56	35	44.69	Medium
Coumarin	22.9	25.19	100	99.99	High
Verapamil	9.7	8.50	98	96.05	High
HBx	-	0.36	-	12.79	Low

Pe: permeability; Fa: absorbed fraction. All of the literature values were cited from C. Zhu et al. Eur. J. Med. Chem. 37 (2002) 399–407 [22].

## Data Availability

Contributions presented in this study are included in the article/Supplementary Materials. Further inquiries can be directed to the corresponding author.

## Supplementary Materials

The following supporting information can be downloaded at: https://www.mdpi.com/article/10.3390/ph18040530/s1, Figure S1: ^1^H NMR spectrum of bixin acquired in a 500 MHz NMR spectrometer in DMSO-d_6_ at 25 °C; Figure S2: ^1^H-^13^C HSQC pureshift spectrum of bixin acquired in a 500 MHz NMR spectrometer in DMSO-d_6_ at 25 °C; Figure S3: ^1^H-^13^C HMBC spectrum of bixin acquired in a 500 MHz NMR spectrometer in DMSO-d_6_ at 25 °C; Figure S4: ^1^H-^1^H COSY spectrum of bixin acquired in a 500 MHz NMR spectrometer in DMSO-d_6_ at 25 °C; Figure S5: ^1^H-^1^H NOESY spectrum of bixin acquired in a 500 MHz NMR spectrometer in DMSO-d_6_ at 25 °C; Table S1: ^1^H NMR, ^1^H-^13^C HSQC pureshift, ^1^H-^13^C HMBC, ^1^H-^1^H COSY and ^1^H-^1^H NOESY (^1^H 500 MHz, ^13^C 125 MHz) chemical shift values of bixin in DMSO-d_6_ (δ in ppm, J values in parentheses) at 25 ºC; Table S2: Validation by redocking of 4IFN. Individual values of each run and RMSD averages for the different functions tested. Software used GOLD v5.4; Table S3: Validation by redocking of 6UO2. Individual values of each run and RMSD averages for the different functions tested. Software used GOLD v5.4.

## Abbreviations

The following abbreviations are used in this manuscript:

ALI	Acute lung inflammation
ARE	Antioxidant responsive element
BAL	Bronchoalveolar lavage
BCA	Bicinchoninic acid
BSA	Bovine serum albumin
COPD	Chronic obstructive pulmonary disease
COSY	Correlation spectroscopy
CSE	Cigarette smoke extract
DAD	Diode-array detector
DAPI	4′,6-Diamidino-2-phenylindole
DMSO	Dimethyl sulfoxide
DMEM	Dulbecco’s Modified Eagle Medium
DNA	Deoxyribonucleic acid
ECL	Enhanced chemiluminescent
EDTA	Ethylenediaminetetraacetic acid
ELISA	Enzyme-linked immunosorbent assay
Fa%	Absorbed fraction
FBS	Fetal bovine serum
FTIR	Fourier transform infrared spectroscopy
GIT	Gastrointestinal tract
GOLD	Genetic Optimization for Ligand Docking
HBx	Bixin
HDAC6	Histone deacetylase 6
HMBC	Heteronuclear multiple bond correlation
HPLC	High-performance liquid chromatography
HSQC	Heteronuclear single quantum coherence
ICAM-1	Intercellular adhesion molecule 1
IL-10	Interleukin-10
IR	Infrared
KC	Keratinocyte-derived cytokine
Keap1	Kelch-like ECH-associated protein 1
KBx	Potassium bixinate
KPE	Potassium-phosphate-buffered saline solution
LDL	Low density lipoprotein
LPS	Lipopolysaccharide
MS	Mass spectrometry
MTT	Methylthiazolyldiphenyl-tetrazolium bromide
NADPH	Nicotinamide adenine dinucleotide phosphate
NF-κB	Nuclear factor kappa-light-chain-enhancer of activated B cells
NMR	Nuclear magnetic resonance
NOS2	Nitric oxide synthase 2
NOESY	Nuclear Overhauser effect spectroscopy
NBT	Nitrotetrazolium blue chloride
Nrf2	Nuclear factor erythroid 2-related factor 2
OD	Optical density
PAHs	Polycyclic aromatic hydrocarbons
PAMPA	Parallel artificial membrane permeability assay
PBS	Phosphate-buffered saline
PDB	Protein Data Bank
Pe	Permeability
PM6	Parametric Method 6
PVDF	Polyvinylidene fluoride
RMSD	Root-mean-square deviation
RNS	Reactive nitrogen species
ROS	Reactive oxygen species
TMS	Tetramethylsilane
TNF-α	Tumor necrosis factor alpha
UMAP	Uniform Manifold Approximation and Projection
