# Supplementary material for: Mechanistic Advances in the Therapeutic Application of Bixin for Lung Inflammation In Vitro and In Vivo
Source: Pharmaceuticals (Basel). 2025 Apr 5;18(4):530. doi: 10.3390/ph18040530 (PMC12030059; doi:10.3390/ph18040530)

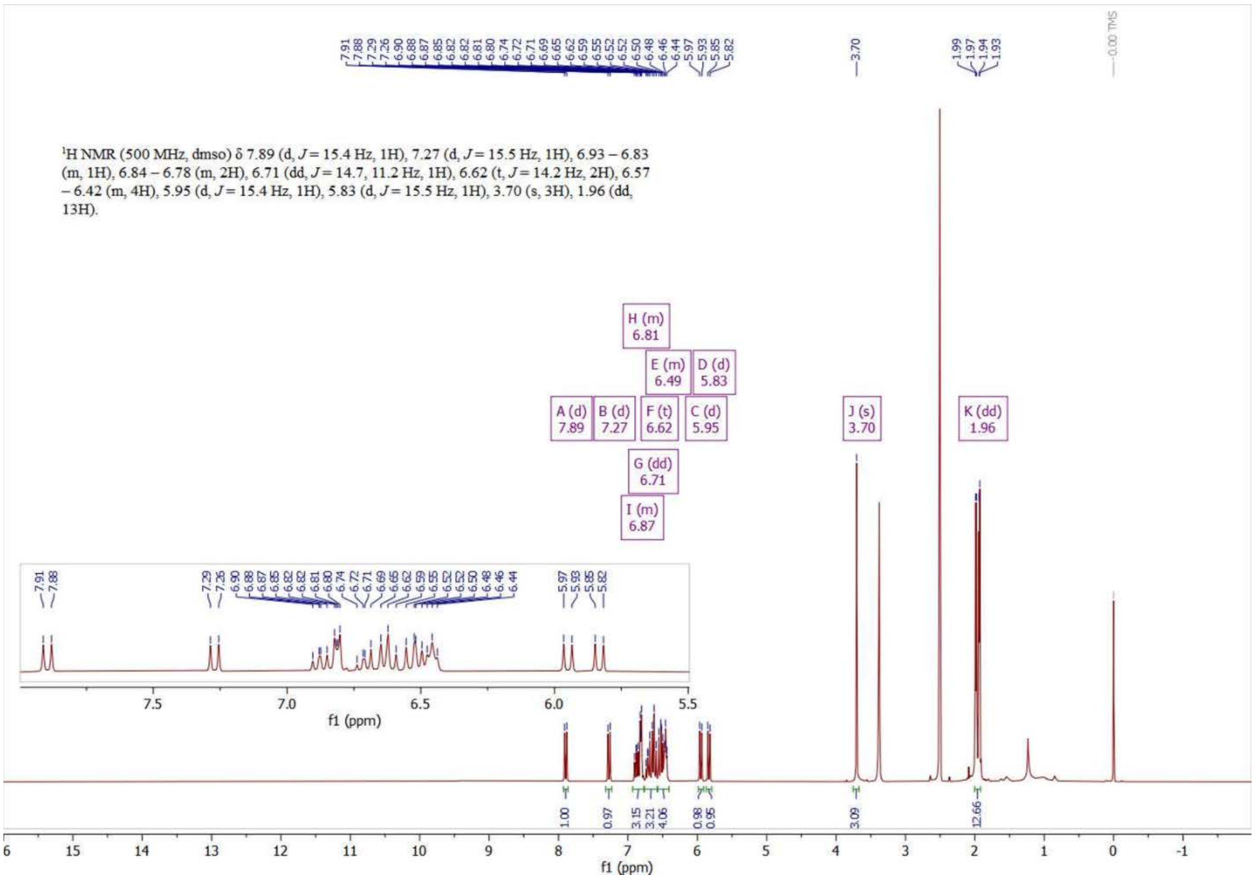

Figure S1. 1H NMR spectrum of bixin acquired in a 500 MHz NMR spectrometer in DMSO-d6 at 25 °C.

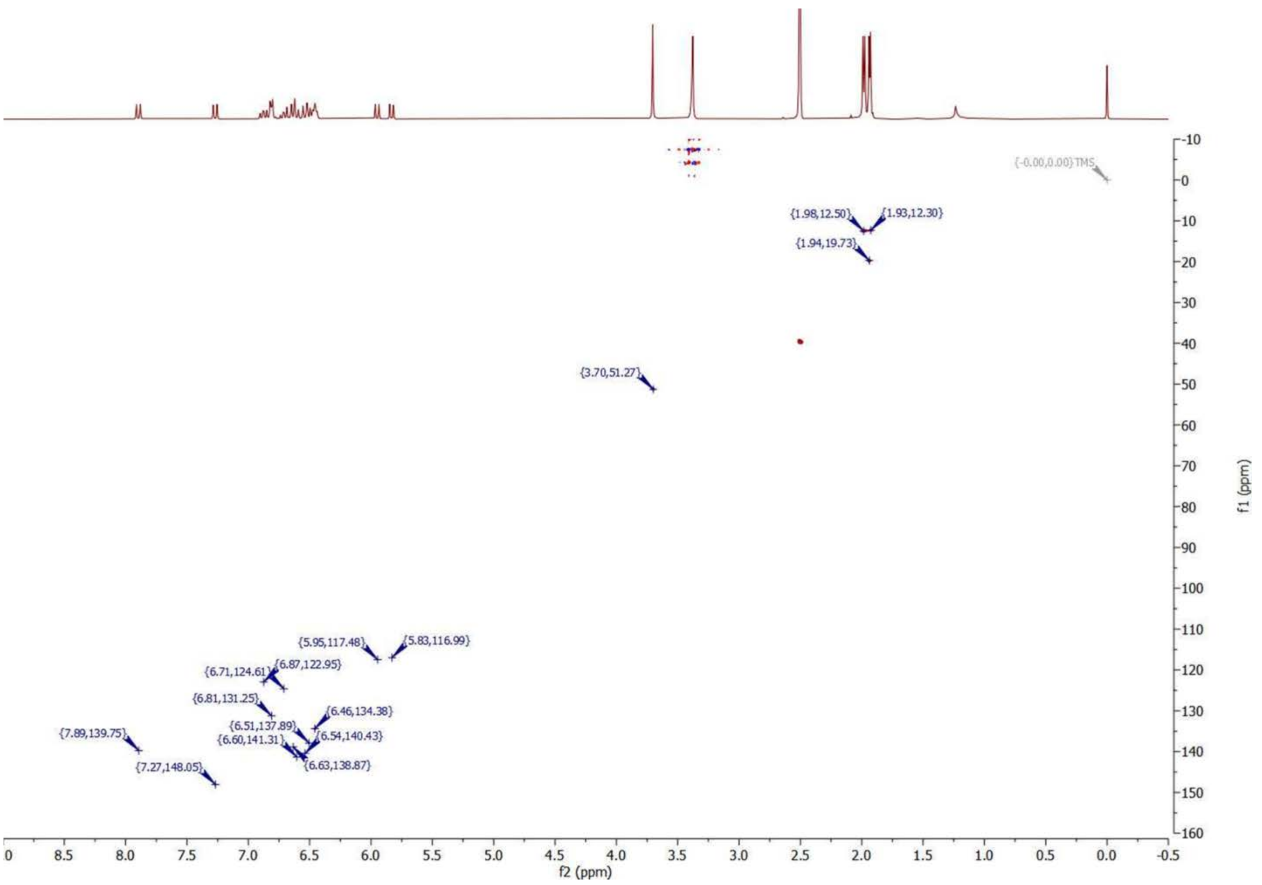

Figure S2. ^1H-^{13}C HSQC pureshift spectrum of bixin acquired in a 500 MHz NMR spectrometer in DMSO-d_6 at 25 °C.

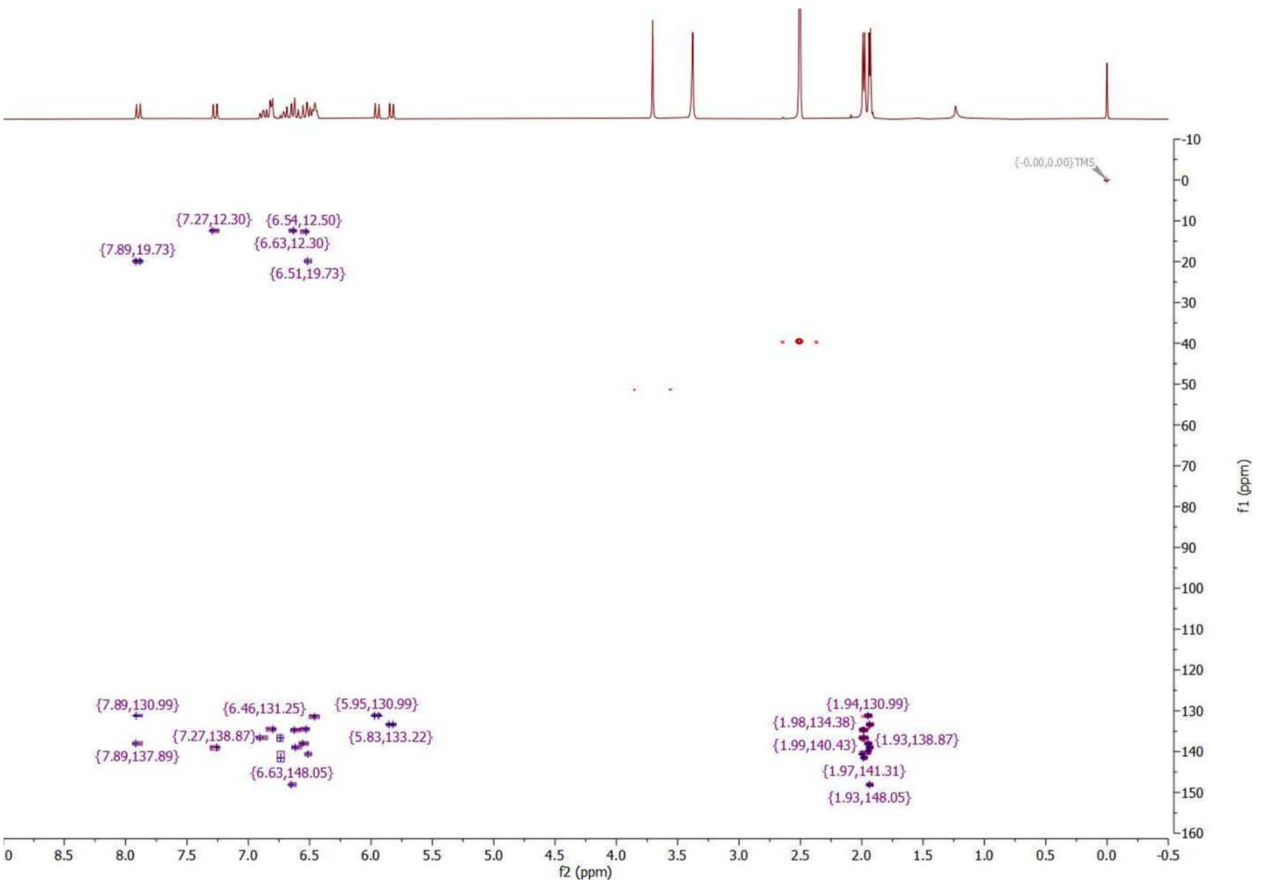

Figure S3. ^{1}H-^{13}C HMBC spectrum of bixin acquired in a 500 MHz NMR spectrometer in DMSO-d_6 at 25 °C.

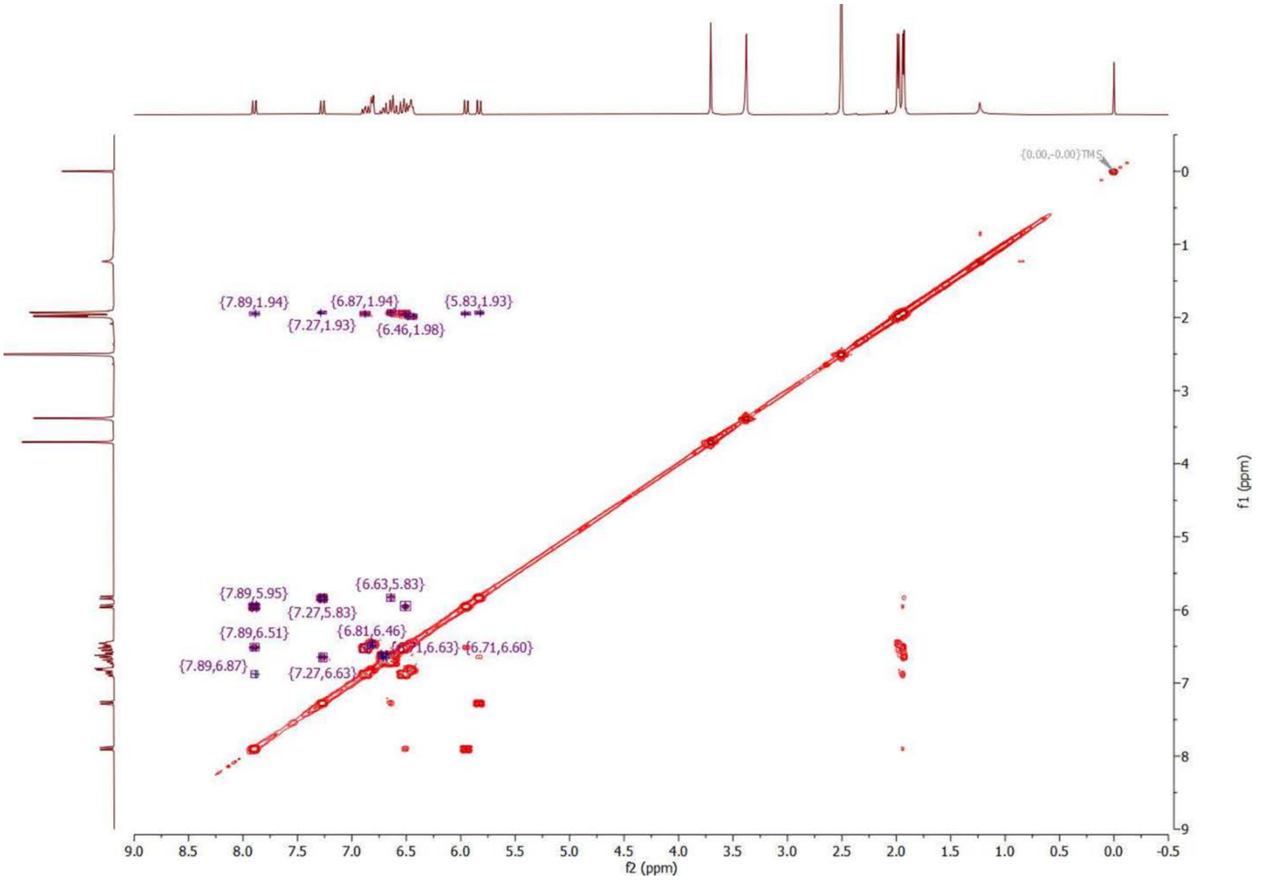

Figure S4. ^{1}H-^{1}H COSY spectrum of bixin acquired in a 500 MHz NMR spectrometer in DMSO-d_6 at 25 °C.

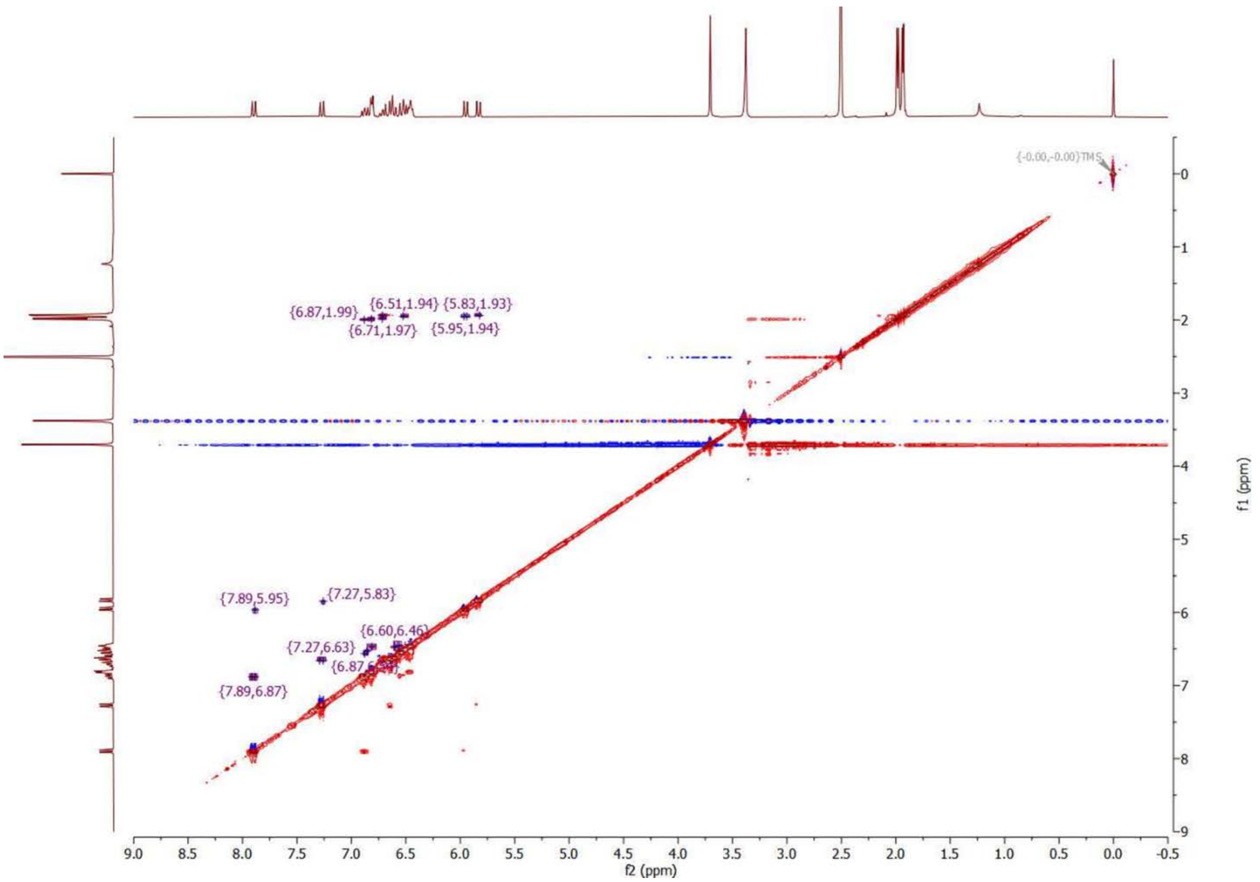

Figure S5. ^{1}H-^{1}H NOESY spectrum of bixin acquired in a 500 MHz NMR spectrometer in DMSO-d$_6$ at 25 °C.

Table S1. ^{1}H NMR, ^{1}H -^{13}C HSQC pureshift, ^{1}H-^{13}C HMBC, ^{1}H-^{1}H COSY and ^{1}H-^{1}H NOESY (^{1}H 500 MHz, ^{13}C 125 MHz) chemical shift values of bixin in DMSO-d$_6$ (δ in ppm, J values in parentheses) at 25 ºC.

**DMSO-*d6***	**HSQC**		**HMBC**	**COSY**	**NOESY**
	**^{1}H**	**^{13}C**			
**1**	-	167.66	-	-	-
**2**	5.83	116.99	133.22<br>167.66	1.93<br>6.63<br>7.27	1.93<br>7.27
**3**	7.27	148.05	12.30<br>138.87<br>167.66	1.93<br>5.83<br>6.63	5.83<br>6.63
**4**	-	133.22	-	-	-
**4’ (CH_3)**	1.93	12.30	133.22	5.83	5.83

			138.87<br>148.05	6.63<br>7.27	6.71
**5**	6.63	138.87	12.30<br>148.05	1.93<br>5.83<br>6.71<br>7.27	7.27
**6**	6.71	124.61	136.62<br>141.31	6.60<br>6.63<br>6.71	1.93<br>1.97
**7**	6.60	141.31	134.38<br>138.87	6.71	6.46
**8**	-	136.62	-	-	-
**8’ (CH_3)**	1.98/1.97	12.50	134.38<br>136.62<br>141.31	6.46	6.71<br>6.81
**9**	6.46	134.38	131.25	6.81	6.60<br>6.81
**10**	6.81	131.25	134.38	6.46	1.98<br>6.46
**11**	6.81	131.25	134.38	6.46	1.98<br>6.46
**12**	6.46	134.38	131.25	6.81	6.54<br>6.81
**13**	-	136.62	-	-	-
**13’ (CH_3)**	1.98/1.99	12.50	134.38<br>136.62<br>140.43	6.46	6.81<br>6.87
**14**	6.54	140.43	12.50<br>134.38<br>137.89	1.98<br>6.87	6.46<br>6.87
**15**	6.87	122.95	136.62	1.94<br>6.51<br>6.54<br>7.89	1.99<br>6.54<br>6.51<br>7.89
**16**	6.51	137.89	19.73<br>140.43	1.94<br>5.95<br>6.87<br>7.89	1.94<br>6.87
**17**	-	130.99	-	-	-
**17’ (CH_3)**	1.94	19.73	130.99<br>137.89<br>139.75	5.95<br>6.51<br>7.89	5.95<br>6.51
**18**	7.89	139.75	19.73<br>130.99<br>137.89<br>166.84	1.94<br>5.95<br>6.51<br>6.87	5.95<br>6.87

**19**	5.95	117.48	130.99<br>166.84	1.94<br>6.51<br>7.89	1.94<br>7.89
**20**	-	166.84	-	-	-
**20’ (OCH_3)**	3.70	51.27	166.84	-	-

Table S2. Validation by redocking of 4IFN. Individual values of each run and RMSD averages for the different functions tested. Software used GOLD v5.4.

**Run**	**ChemScore**	**ChemPLP**	**GoldScore**	**ASP**
1	6.757	2.717	2.879	8.542
2	7.759	7.897	2.615	8.395
3	7.157	5.739	0.960	6.849
4	6.230	4.422	2.807	5.231
5	6.146	5.704	1.356	8.257
6	7.553	5.417	2.742	2.629
7	6.266	6.027	1.038	7.846
8	7.514	7.977	2.787	7.647
9	6.198	7.784	2.241	5.649
10	7.638	5.699	2.169	5.831
**Average**	**6.922**	**5.938**	**2.159**	**6.687**

Table S3. Validation by redocking of 6UO2. Individual values of each run and RMSD averages for the different functions tested. Software used GOLD v5.4.

Run	ChemScore	ChemPLP	GoldScore	ASP
1	0.9692	0.9004	1.0476	0.9526
2	1.4331	1.0074	0.8922	0.9492
3	1.0605	0.8744	1.0752	1.0127
4	0.9182	1.0846	0.9966	1.4635
5	8.6747	1.2769	0.9609	1.2322
6	1.1587	0.8703	1.7897	0.9901
7	1.3858	0.9577	0.9033	1.1358
8	0.9868	0.9539	1.1343	0.7881
9	1.1531	0.9323	0.8677	1.0311
10	1.0781	1.2403	1.2043	1.0131
**Average**	**1.88182**	**1.00982**	**1.08718**	**1.05684**

Unedited and uncropped full image western blots – first sample

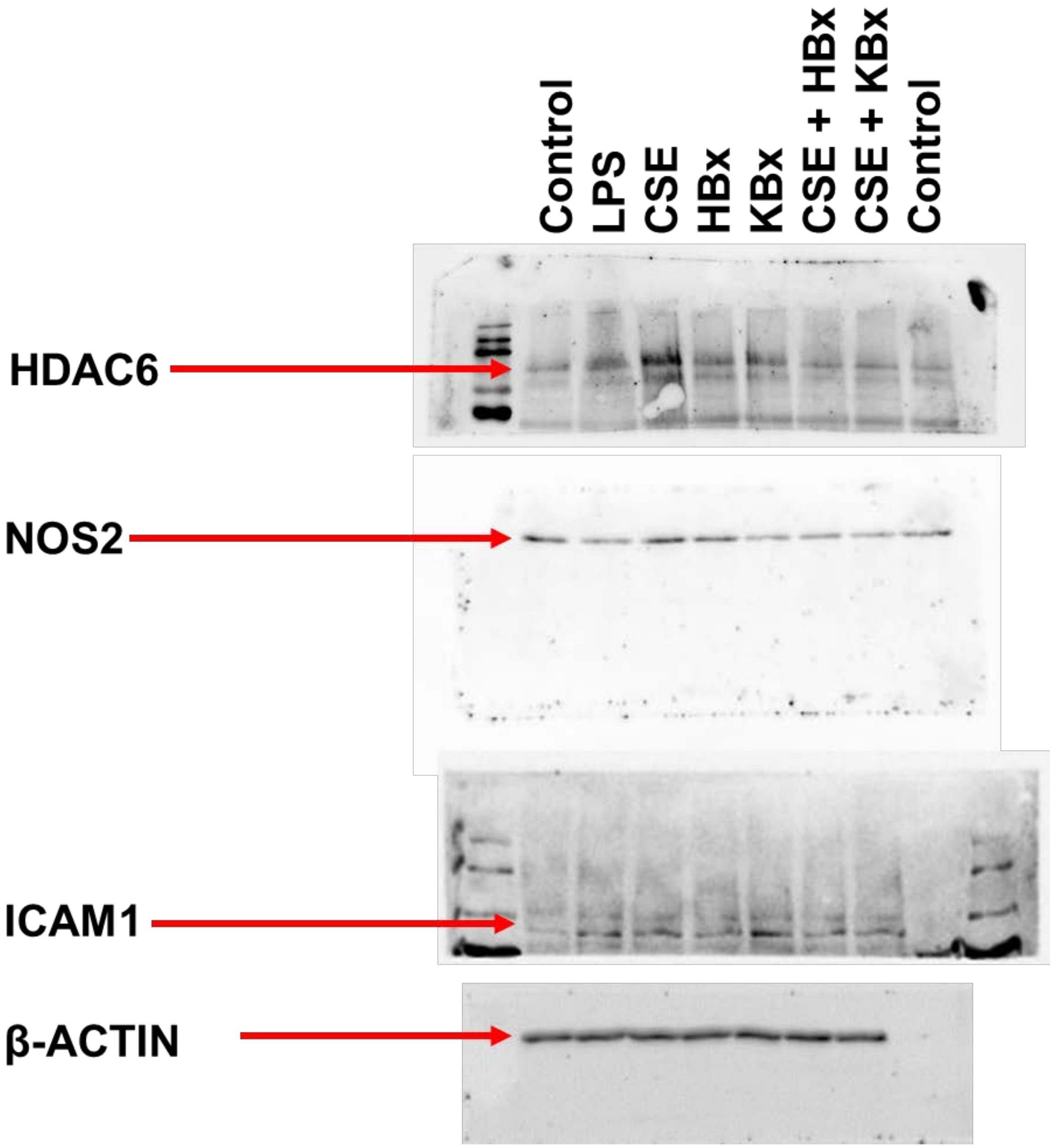

Unedited and uncropped full image western blots – second sample

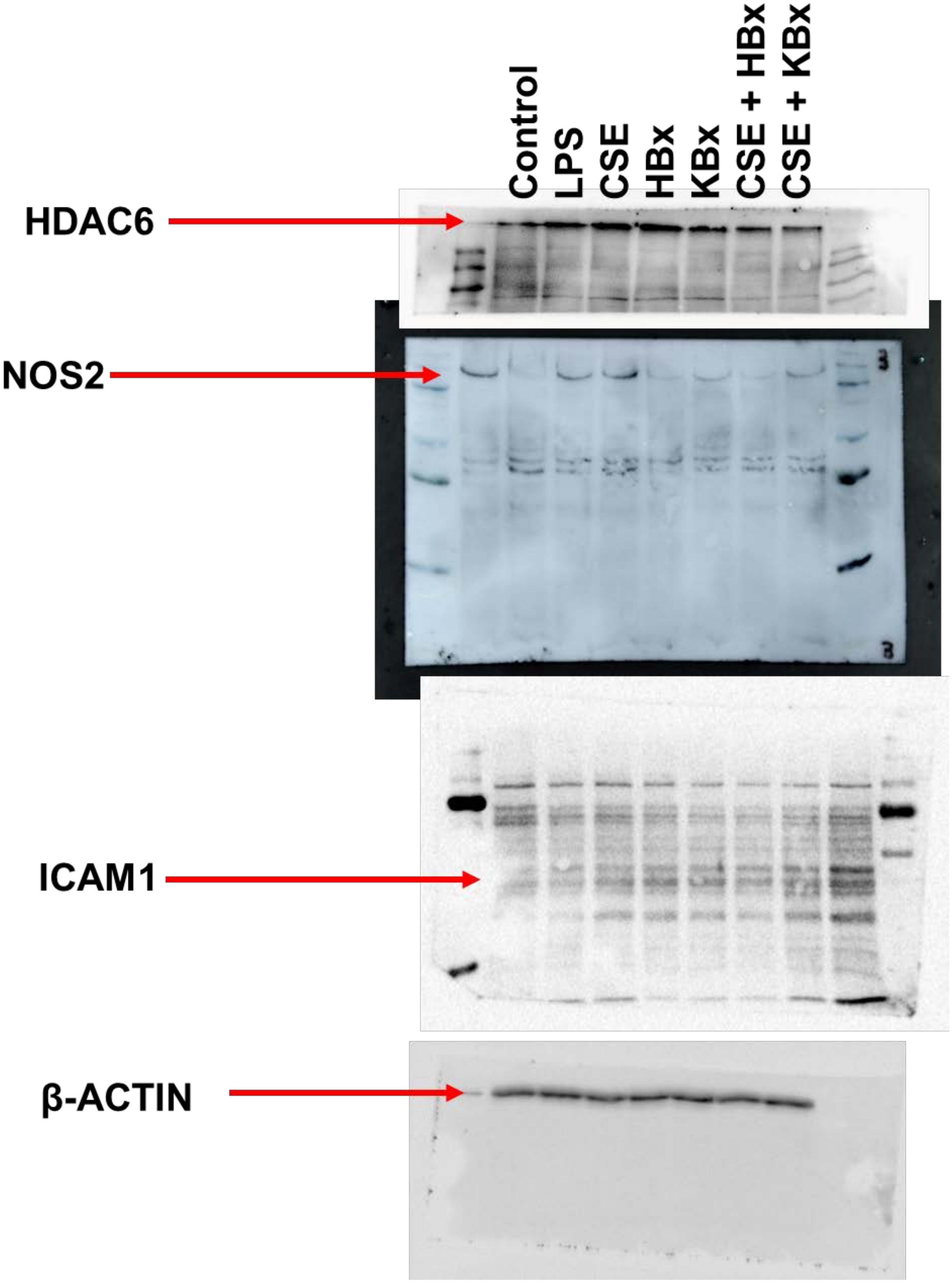

Unedited and uncropped full image western blots – third sample

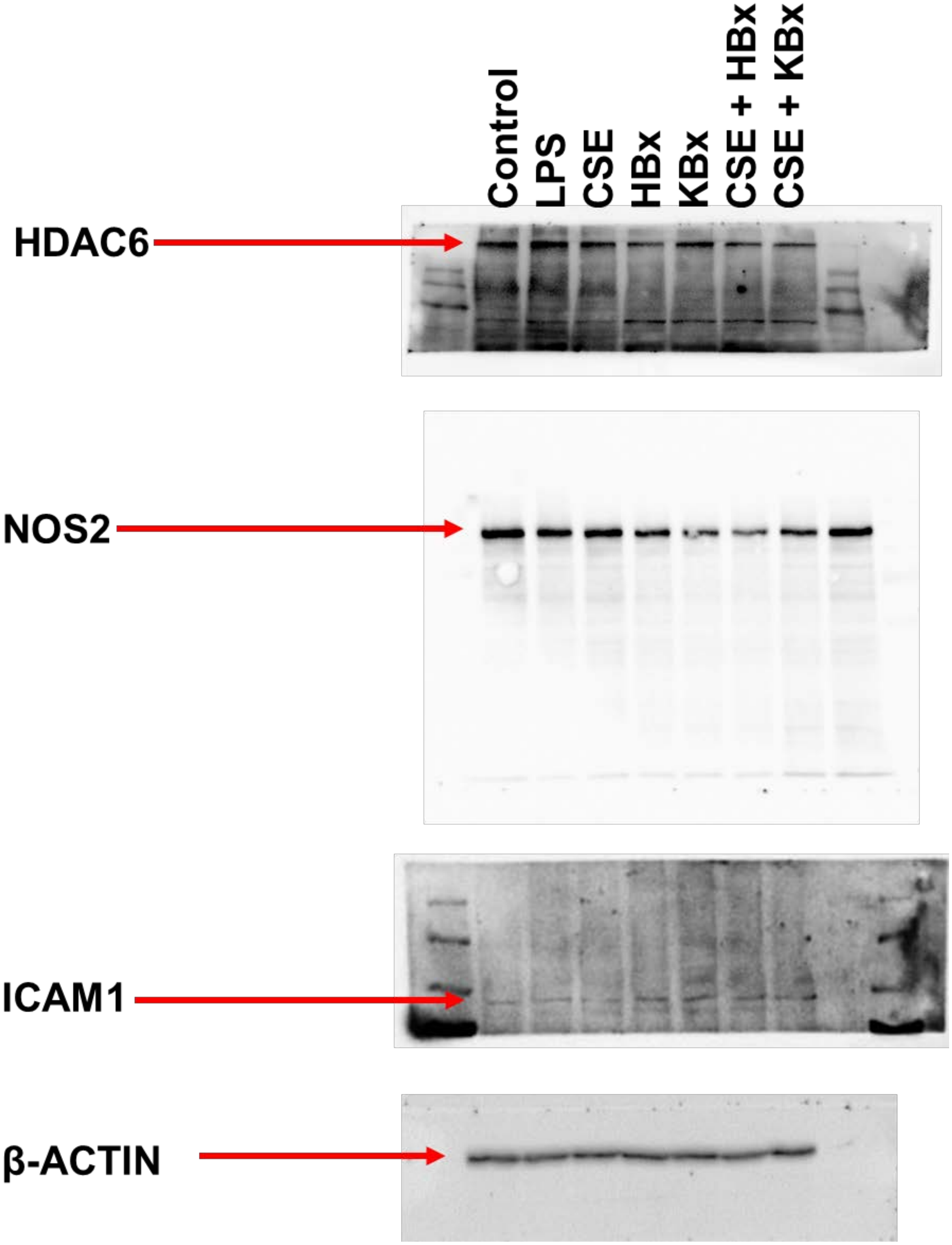

Supplement: Supplementary file 1 [file pharmaceuticals-18-00530-s001.zip › pharmaceuticals-3556280-supplementary.pdf]
